# Heterolayered Carbonized MXene/Polyimide Aerogel for Low-Reflection Electromagnetic Interference Shielding and Multi-Spectrum Compatible Protection

**DOI:** 10.1007/s40820-025-02027-1

**Published:** 2026-01-13

**Authors:** Shan Zhang, Chen-Ming Liang, Lu Zhou, Juntao Wu, Martin C. Koo, Zongxin Wu, Yun-Tian Chen, Guang-Sheng Wang

**Affiliations:** 1 State Key Laboratory of Bioinspired Interfacial Materials Science, Bioinspired Science Innovation Center, Hangzhou International Innovation Institute, Beihang University, Hangzhou, 311115 People’s Republic of China; 2 School of Chemistry, Beihang University, Beijing, 100191 People’s Republic of China

**Keywords:** Heterolayered, Electromagnetic interference shielding, Multi-spectrum, Low reflection, High efficiency

## Abstract

The advancement of next-generation high-frequency communication systems and stealth detection technologies necessitate the development of efficient, multi-spectrum compatible shielding materials. However, the achievement of simultaneous high efficiency and low reflectivity across microwave, terahertz, and infrared spectra remains a formidable challenge. Herein, a carbonized MXene/polyimide (C-MXene/PI) aerogel material integrating a spatially coupled hierarchically anisotropic structure with stepwise conductivity gradients was constructed. Electromagnetic waves propagate through the top-down vertical disordered horizontal architecture and progressive conductivity gradient of C-MXene/PI aerogel, undergoing stepwise absorption–dissipation–re-dissipation processes. The C-MXene/PI aerogel exhibits an average electromagnetic interference (EMI) shielding effectiveness of 91.0 dB in X-band and a reflection coefficient of 0.40. In the terahertz frequency band, the average EMI shielding performance reaches 66.2 dB with a reflection coefficient of 0.33. Furthermore, the heterolayered porous architecture of C-MXene/PI aerogels exhibits low thermal conductivity and reduced infrared emissivity, enabling exceptional infrared stealth capability across the 2–16 μm wavelength spectrum. This study provides an feasible strategy for constructing low-reflectivity multi-spectrum compatible shielding materials.

**Supplementary Information:**

The online version contains supplementary material available at 10.1007/s40820-025-02027-1.

## Highlights

- A carbonized MXene/polyimide (C-MXene/PI) aerogel with hierarchically anisotropic and gradient electrical conductivity structures was constructed via a stepwise freezing strategy.
- The C-MXene/PI aerogel shows a high EMI shielding effectiveness of 91.0 dB with a low reflection coefficient of 0.40 in the X-band, alongside a high shielding of 66.2 dB with an excellent low reflection of 0.33 in the THz band.
- The C-MXene/PI aerogel exhibits low thermal conductivity and reduced infrared emissivity, enabling exceptional infrared stealth capability across the 2–16 μm wavelength spectrum.

**Supplementary Information:**

The online version contains supplementary material available at 10.1007/s40820-025-02027-1.

## Graphical abstract

**Figure:**
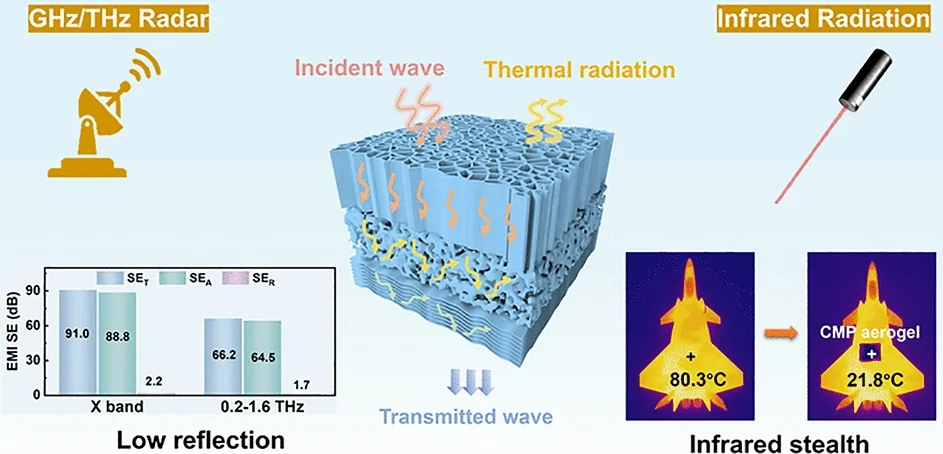

## Introduction

In applications such as radar stealth, aerospace platform protection, and the protection of critical facilities/equipment from electronic warfare, there exists an urgent need to develop electromagnetic protective materials applicable across the microwave, terahertz, and infrared bands [1–4]. Materials with high electrical conductivity, such as metal-based materials (silver/copper foil), emerging two-dimensional materials (MXene films), or carbon-based macrostructures (graphene foam), typically exhibit high electromagnetic interference shielding effectiveness in the microwave and terahertz frequency bands, coupled with low infrared emissivity, thereby offering excellent stealth performance. However, their significant impedance mismatch often leads to strong electromagnetic wave reflection [5–8]. This not only increases the risk of secondary pollution but also renders it vulnerable to radar detection systems. Therefore, the development of protective materials with multi-spectral compatibility, high shielding efficiency, and low reflection represents a pivotal goal for the future advancement of stealth materials.

Porous materials demonstrate significant potential as high-performance multi-spectrum compatible protection materials due to their unique characteristics of low density, high specific surface area, and tunable impedance matching [9–11]. In particular, high porosity facilitates multiple scattering and attenuation of electromagnetic waves, offering a pathway to achieving low reflection characteristics. However, traditional homogeneous porous materials typically require a high content of conductive fillers or denser structures to attain sufficient electromagnetic interference shielding effectiveness (SE) [12]. This approach inevitably exacerbates electromagnetic wave reflection (resulting in a high reflection coefficient, R) [13, 14]. Conversely, pursuing low reflection often compromises shielding effectiveness [15, 16]. This inherent contradictory relationship between high SE and low R complicates the attainment of effective multi-band shielding in homogeneous porous materials.

The design of multilayered structures is recognized as an effective strategy for developing low reflection EMI shielding materials. For instance, asymmetric or layered configurations have been engineered to guide electromagnetic waves through an absorption–reflection–reabsorption process, significantly reducing reflection while maintaining high SE [17]. Moreover, constructing gradient conductive pathways within multilayered architectures enables superior impedance matching, effectively minimizing reflection loss [18]. Additionally, the combination of structural design and low-emissivity materials can achieve efficient thermal insulation and infrared thermal camouflage [19–22]. However, current research primarily focuses on single-band applications such as microwave or infrared spectra, lacking comprehensive exploration of materials capable of simultaneous electromagnetic dissipation across microwave, terahertz, and infrared bands. Therefore, overcoming the limitations of conventional homogeneous structures and designing novel aerogel materials with multi-spectral compatibility across microwave, terahertz, and infrared bands based on electromagnetic loss mechanisms in different porous frameworks, while maintaining high electromagnetic interference shielding effectiveness and low reflection coefficients, remains a significant challenge in current research.

Herein, we report an effective strategy for constructing aerogels with hierarchical anisotropy and graded electrical conductivity structures via stepwise freezing. The C-MXene/PI aerogel features a tri-layered anisotropic structure: radial electromagnetic wave (EMW) incidence layer, disordered EMW dissipation layer, and horizontal lamellar reflection layer. An electrical conductivity gradient is formed by gradually increasing the MXene content from the radial layer to the horizontal layer. Electromagnetic waves undergo an “absorption–dissipation–re-dissipation” process in the multi-level anisotropic and conductive gradient structure of the aerogel. As a result, C-MXene/PI aerogel demonstrates a high EMI SE of 91.0 dB and a low reflection coefficient (R = 0.40) in the X-band, while also maintaining excellent low-reflection (R = 0.33) and high EMI shielding performance (66.2 dB) in the terahertz band. Furthermore, the porous structure C-MXene/PI aerogel exhibits a low infrared emissivity and low thermal conductivity, while demonstrating excellent infrared stealth performance in the 2–16 μm wavelength range. This work provides a novel strategy for designing multi-spectrum compatible protective materials with low reflectivity and high performance, demonstrating its potential for widespread application in military and aerospace sectors.

## Methods

### Main Materials

4,4′-oxydianiline (ODA; 99%), Pyromellitic dianhydride (PMDA, 99%) was purchased from Shanghai Research Institute of Synthetic Resins and sublimated prior to use. Ti_3_AlC_2_ powders (99%, 400 mesh) were purchased from Jilin 11 technology Co., Ltd. Lithium fluoride (LiF, 99%). Hydrochloric acid (HCl, 37 wt%), N, N-dimethyl-formamide (DMF, analytically pure, ≥ 99.5%) and triethylamine (TEA, 99.0%) were supplied by Beijing Chemical Works.

### Synthesis of Ti_3_C_2_T_x_ MXene Flakes

Few-layer Ti_3_C_2_T_x_ flakes were synthesized by selective etching of Ti_3_AlC_2_ powder using LiF/HCl solvent. Firstly, 7.5 mL of deionized water was added to 22.5 mL of analytically HCl (37.0 wt%) and diluted to obtain a hydrochloric acid solution with a concentration of 9 M. Then the prepared hydrochloric acid solution was added to a 100 mL of polytetrafluoroethylene reactor, followed by the addition of 1.6 g of LiF, and stirred for 30 min. Then 1 g Ti_3_AlC_2_ was slowly added to the above solution and stirred continuously at 35 °C for 24 h. The product was washed with deionized water and centrifuged at 3500 rpm. Finally, the few-layer Ti_3_C_2_T_x_ MXene dispersion solution was obtained by ultrasonication under the protection of argon gas stream for 1 h, followed by centrifugation at 3500 rpm for 20 min. The obtained low-layer MXene solution was centrifuged at 11,000 rpm for 30 min at high speed to obtain concentrated Ti_3_C_2_T_x_ condensation, and then the MXene condensation was diluted with water into 40 mg mL^−1^ MXene solution. The characterizations of MXene nanosheets are shown in Fig. S1.

### Preparation of the C-MXene/PI Aerogel with Different Structures

A measured amount of ODA was added to a three-necked flask and dissolved in an appropriate amount of DMF. After complete dissolution of the ODA monomer, PMDA was added in three portions. The mixture was stirred and reacted for 6 h. The resulting solution was then poured into deionized water for precipitation. The precipitate was vacuum-dried and ground into polyamic acid (PAA) powder. 0.3 g of the above powder was dissolved in a mixture of 0.096 g TEA and 29.304 g deionized water, yielding an aqueous solution of the water-soluble polyimide (PI) precursor, termed the poly(amic acid) salt (PAS) solution. The synthesis process of PAS is illustrated in Fig. S2.

2.7 mL of MXene solution (10 mg mL^−1^) was added to the 30 mL PAS solution (1 wt%) and mixed uniformly, yielding the MXene/PAS solution. Vertically Structured Aerogel: The PAS solution was placed in a unidirectional freezing mold with a copper plate at the bottom. It was frozen in liquid nitrogen and subsequently freeze-dried. Disordered Structured Aerogel: The PAS solution was placed in a polytetrafluoroethylene (PTFE) mold without a copper plate. It was frozen in a refrigerator (-20 °C) and subsequently freeze-dried. Horizontal Structured Aerogel: The PAS solution was placed in a unidirectional freezing mold with copper plates on the sides. It was frozen in liquid nitrogen and subsequently freeze-dried.

Heterolayered structure MXene/PAS aerogel: The 30 mL of PAS solution (1 wt%) was equally divided into three portions. 0.9 mL of MXene solution (10 mg mL^−1^) was added to the 10 mL PAS solution (1 wt%) and mixed uniformly, yielding the MXene/PAS solution. The first portion was transferred into a custom mold and frozen in liquid nitrogen for 30 min. After complete solidification of the first layer, the mold containing the frozen layer was placed in a refrigerator to stabilize the sample temperature uniformly. The second portion was then added onto the frozen first layer and frozen in the refrigerator for 3 h (Fig. S3). Finally, after the second layer was fully solidified, the third portion was added onto the second layer, placed in the custom-made mold, and frozen in liquid nitrogen for 30 min. After freeze-drying, a multilayer structure MXene/PAS aerogel was obtained. Multiple repeated experiments confirmed that the prepared samples exhibited consistent structural integrity and performance.

Conductivity-Gradient Aerogel: The 30 mL PAS solution (1 wt%) was divided equally into three portions. To these portions, 0.5, 0.8, and 1.5 mL of MXene solution (10 mg mL^−1^) were added, respectively, yielding mixed MXene/PAS solutions with PAS:MXene ratios of 20:1, 12.5:1, and 6.7:1. Then, the MXene/PAS solutions mixed in a ratio of 20:1, 12.5:1, and 6.7:1 were successively frozen into the first, second and third layers of multilayer aerogels using the aforementioned method. After freeze-drying, the heterolayered MXene/PAS aerogels with a conductivity gradient structure (MXene/PAS-BG) were obtained.

MXene/PAS aerogels were heat-treated in N_2_ atmosphere (gradient temperature rise: 80 °C, 30 min; 120 °C, 30 min; 180 °C, 30 min; 250 °C, 30 min; 300 °C, 30 min; 350 °C, 30 min), yielding MXene/PI (MP) aerogels. The MXene/PI aerogels were then carbonized in a furnace under an inert gas atmosphere at 1100 °C for 2 h, yielding C-MXene/PI aerogels (CMP). The C-MXene/PI aerogel monolith with dimensions of 1 cm × 2 cm × 1 cm (volume: 2 cm^3^) was prepared, exhibiting a mass of 24 mg.

### Statistics and Reproducibility

The reproducibility of the synthesis process for the heterolayered C-MXene/PI aerogel was rigorously evaluated through five independent experimental replicates conducted under identical conditions. The resulting structural morphology and key functional properties including EMI shielding effectiveness in the X-band and 0.2–1.6 THz range reflection coefficient and thermal conductivity were confirmed to be highly consistent and reproducible across all repeated experiments. The data from these five comparative batches are presented in Table S5.

### Characterizations

The morphology was observed by a scanning electron microscope (SEM, Quanta 250 FEG and JEOL, JSM-7500F). The transmission electron microscopy (TEM) images of MXene flakes were recorded by field-emission transmission electron microscopy (FETEM, JEM-2100F). The crystal structure was investigated by powder X-ray diffraction (XRD, Rigaku Ultima IV). The surface element and element valence states were investigated by X-Ray Photoelectron Spectroscopy (XPS, Thermo Scientific K-Alpha). The chemical bonds of the composite films were analyzed via a Fourier transform infrared spectroscopy (FTIR, Nicolet iS50, Thermo Fisher Scientific, Waltham, MA). The height morphology of MXene flakes was characterized by the atomic force microscope (AFM, dimension icon, Bruker). The density of the C-MXene/PI aerogel were measured by weighing films with precise dimensions. Five parallel measurements of density were performed on each series of samples. The electrical conductivity of the aerogel was measured using a four-probe conductivity meter (KDB-3, dual-combination). The conductivity can be calculated based on the resistance measured by the four-probe conductivity meter and the thickness measured by the ruler. The formula is as follows:

1 $$\sigma = \frac{1}{R \times t}$$

where R is the resistance, and t is the thickness of the sample. The R and t values of the C-MXene/PI aerogel are presented in Table S6.

The EMI shielding performance of the composite films was tested using an Agilent PAN-N5244A vector network analyzer via the waveguide method (X band). The measured scattering parameters (S_11_ and S_21_) were used to calculate the absorption, reflection, and total shielding value of the samples. EMI SE (SE_T_) was divided into three parts: reflection loss (SE_R_), absorption loss (SE_A_), and multiple reflection loss (SE_MR_). EMI SE, R, and A were calculated from the S parameters according to the following equations:

2 $${\mathrm{R}} = \left| {S_{11} } \right|^{2}$$

3 $${\mathrm{A}} = {1} - \left| {S_{11} } \right|^{2} - \left| {S_{21} } \right|^{2}$$

4 $${\mathrm{SE}}_{{\mathrm{R}}} = - {1}0\;{\mathrm{lg}}\left( {{1} - \left| {S_{11} } \right|^{2} } \right)$$

5 $${\mathrm{SE}}_{{\mathrm{A}}} = - {1}0\;{\mathrm{lg}}\left( {\frac{{\left| {S_{21} } \right|^{2} }}{{1 - \left| {S_{11} } \right|^{2} }}} \right)$$

6 $${\mathrm{EMI}}\;{\mathrm{SE}} = {\mathrm{SE}}_{{\text{R + }}} {\mathrm{SE}}_{{\mathrm{A}}}$$

We calculated the Specific Shielding Effectiveness (SSE), which is defined as SSE = Total Shielding Effectiveness (SE_T_) / (material density × thickness).

The reflective and transmissive properties were investigated by a terahertz time-domain spectrometer system (TPF 15 K, Terahertz Photonics, China). The infrared reflectivity, transmissivity, and emissivity were revealed by a dual-band emissivity meter (IR-2, Shanghai Chengbo Optoelectronic Technology, China) in the wavelength range of 1–22 μm. The thermal stability was investigated by a thermal analyzer (Netzsch, STA449F3). Thermal imaging camera was used to record the temperature of composite films in the photothermal experiment (ST9450). The thermal conductivity of heterolayered aerogels was characterized based on the transient plane heat source method (Hot Disk TPS2500S). The infrared emissivity of MP and CMP was revealed by a dual-band emissivity meter (IR-2, Shanghai Chengbo Optoelectronic Technology, China) in the wavelength range of 2–16 µm.

### CST Simulation of C-MXene/PI Aerogel

The electromagnetic simulations were conducted using CST Studio Suite 2014. The current density distribution, electric field distribution, and power loss distribution of the C-MXene/PI aerogel were simulated by importing material models and incorporating electrical conductivity parameters, with specific conductivity values provided in Table S7. To optimize computational efficiency, periodic boundary conditions were applied to a single unit cell. The electromagnetic source was conFig.d with periodic boundary conditions parallel to the material surface and open boundary conditions with additional space perpendicular to the material surface. Mesh convergence was achieved through adaptive mesh refinement using hexahedral mesh elements.

## Results and Discussion

### C-MXene/PI aerogel Preparation and Structure Characterization

**Fig. 1:**
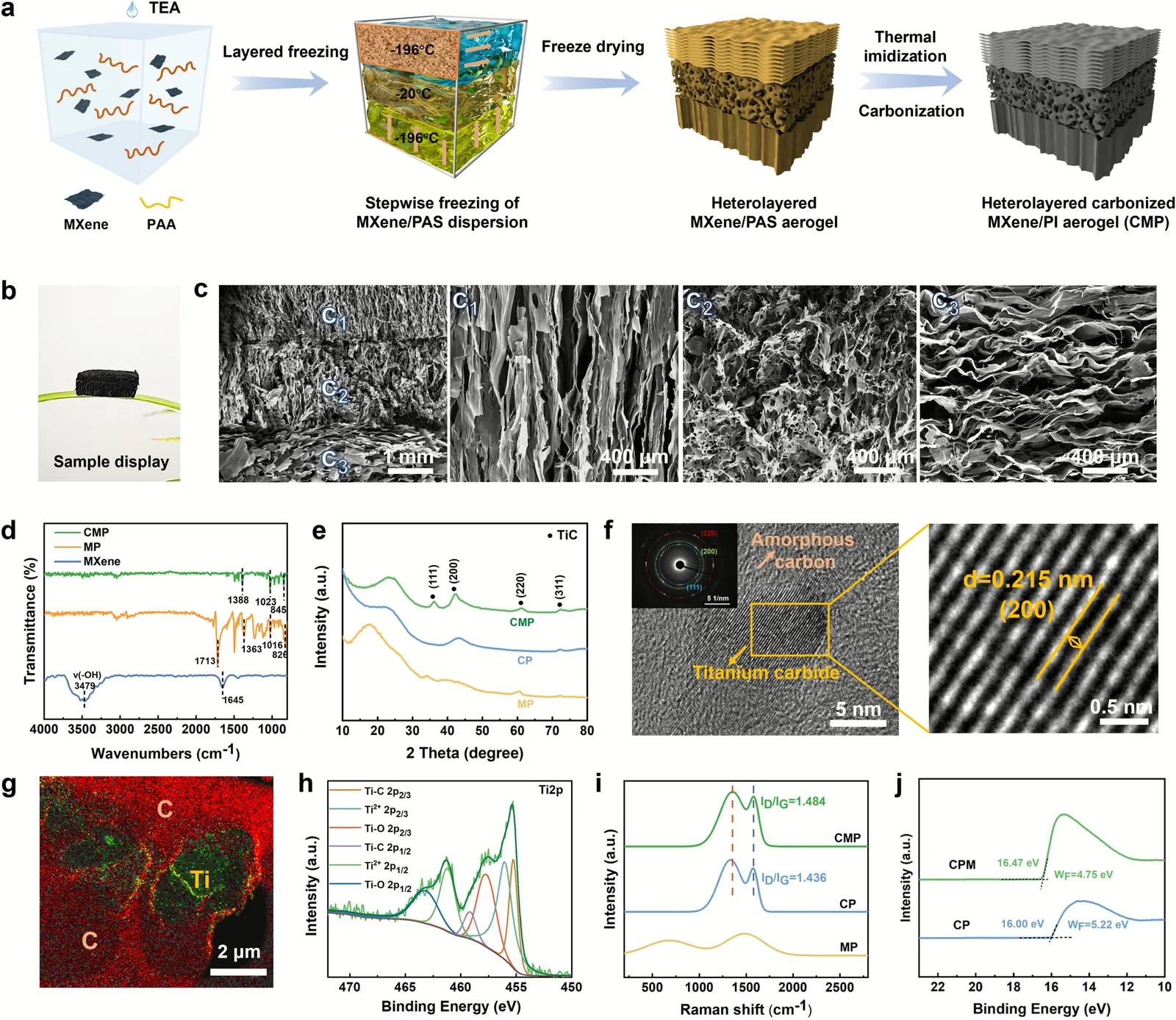
The fabrication process of the heterolayered CMP aerogel with electrically conductive gradient structure and morphology characterization. **a** Diagram of the device used for stratified stepwise freezing-drying. **b** Optical image demonstrating the lightweight of CMP aerogel. **c** The cross-sectional-SEM images of heterolayered CMP aerogel. (c_1_) The first layer-vertical structure. (c_2_) The second layer-disordered structure. (c_3_) The third layer-horizontal structure. **d** FT-IR spectrum of MXene, MP and CMP. **e** High-throughput XRD spectra of MP, CP and CMP. **f** HRTEM image of the TiC-C interface. The top-left inset shows the SAED pattern of TiC, and the magnified view reveals the lattice fringes of the TiC (200) plane. **g** TEM-EDS elemental mapping of CPM. **h** High-resolution XPS spectra of Ti 2*p* for CMP. **i** Raman spectra of MP, CP and CMP. **j** UPS spectra of CP and CMP

Figure 1 illustrates the fabrication process of the CMP. The tri-heterolayered MXene/poly(amide acid) ammonium salt (PAS) aerogels were fabricated via layered freezing of MXene/poly(amide acid) PAA/ triethylamine (TEA) solution followed by freeze-drying. The layered freezing process was implemented in a self-made freezing mold (Fig. S3). In detail, by freezing MXene/PAS dispersions under vertical temperature gradients (− 196 °C), uniform temperature gradients (− 20 °C), and horizontal temperature gradients (− 196 °C), respectively, the first layer with a vertical pore structure, the middle layer with a disordered pore structure, and the third layer with a layered horizontal structure were sequentially constructed. Additionally, the amount of MXene added gradually increased from the first layer (vertical) to the middle layer (disordered) and then to the third layer (horizontal) in sequence (Figs. S4 and S5). Subsequently, the frozen heterolayered MXene/PAS aerogel was freeze-dried to remove the ice crystal templates and thermally annealed to achieve the polymerization of the polyimide (PI) macromolecules. After drying, the MXene/PI aerogel (MP) was subjected to high-temperature treatment in a tube furnace, where co-carbonization was carried out at 1100 °C, resulting in the formation of the 3D heterogeneous structural network. The synthesized heterolayered carbonized MXene/PI aerogel (CMP) possesses an ultralow density of approximately 12 mg cm⁻^3^, being sufficiently lightweight to rest on a leaf while demonstrating exceptional mechanical strength by supporting a 500 g weight (Figs. 1b and S6).

The cross-section morphology in the axial direction of CMP is presented in Fig. 1c, where distinct orientation differences were observed among the tri-layer structures. From the radial cross-sectional morphology of CMP layers, the first layer demonstrates vertically oriented vertical growth structures (Fig. 1c1), the intermediate transitional zone manifests as a disordered porous architecture (Fig. 1c2), and terminal stratum features horizontally aligned development configurations (Fig. 1c3). As shown in Figs. S7 and S8, the SEM images demonstrate that the three layers are closely attached at the interface and will not separate even when twisted. To elucidate the interfacial interaction mechanisms between MXene and PI during high-temperature co-carbonization, systematic chemical-structural characterization was performed with multiscale analytical protocols. FTIR analysis revealed that MP exhibited distinct characteristic peaks at 1713, 1363, and 1016 cm^−1^. After undergoing high-temperature carbonization, the absorption peaks of CMP disappeared or weakened, indicating that MP was partially transformed into amorphous carbon (Fig. 1d). The high-throughput XRD pattern clearly shows a distinct broad peak at 23.8° for CMP, corresponding to the amorphous carbon formed after carbonization of PI. Clear titanium carbide (TiC) peaks are observed near 36.1°, 42.1°, 61.2°, and 72.3°, which are assigned to the (111), (200), (220), and (311) crystal planes of TiC (Fig. 1e). HRTEM images reveal lattice fringes with spacings of d = 0.25 nm and d = 0.215 nm, matching the (111) and (200) planes of TiC (Figs. 1f and S9). The SAED pattern displays distinct polycrystalline diffraction rings corresponding to the (111), (200), and (220) planes of TiC (Fig. 1f). TEM-EDS and XPS spectra clearly detect elements such as C and Ti in CMP (Fig. 1g and S10). The Ti 2*p* spectrum exhibits five main peaks at 455.3, 456.0, 457.8, 459.3, 461.2, and 463.2 eV, corresponding to Ti-C 2*p*_3/2_, Ti^2^⁺ 2*p*_3/2_, Ti–O 2*p*_3/2_, Ti-C 2*p*_1/2_, Ti^2^⁺ 2*p*_1/2_, and Ti–O 2*p*_1/2_ bonds, respectively (Fig. 1h). These results indicate that the high-temperature co-carbonization of PI and MXene leads to a more pronounced transformation of MXene into TiC, accompanied by a significant enhancement in Ti-C bonding strength.

Raman spectroscopy reveals prominent D and G bands in carbonized CMP. Compared to MXene-free carbonized PI, CMP exhibits a higher I_D_/I_G_ ratio (1.484), indicating that the decomposition of functional groups on MXene during co-carbonization introduces additional defects within the 3D heterogeneous network (Fig. 1i). The ultraviolet photoelectron spectroscopy (UPS) results show that compared with CP, the Fermi level of CMP increased by 0.47 eV, from − 5.22 to − 4.75 eV, after doping with carbonized MXene (Fig. 1j). This clearly demonstrates charge transfer during the MXene doping process, which elevates the Fermi level of CMP and substantially enhances its electrical conductivity. The above results confirm that the high-temperature co-carbonization of PI and MXene forms amorphous carbon and TiC crystalline phases. The introduction of MXene improves the electrical conductivity of the co-carbonized material, which positively influences the electromagnetic interference shielding performance of the composite.

### Electromagnetic Shielding Performance of C-MXene/PI aerogel and the Low Reflection Mechanism

**Fig. 2:**
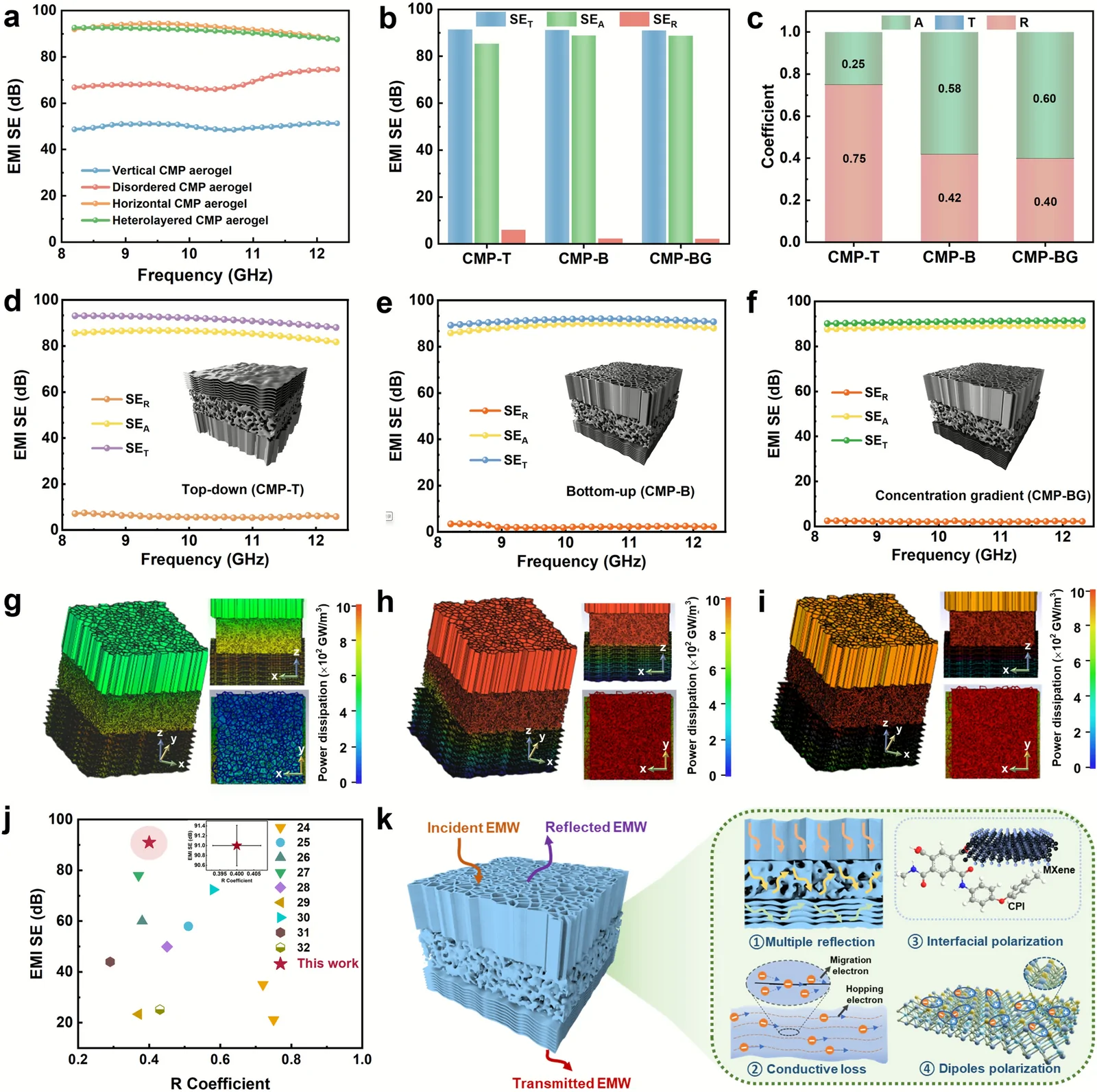
The EMI shielding performances and simulated results of CMP. **a** EMI shielding performances of different structural CMP aerogels, **b** SE_T_, SE_A_ and SE_R_ average value and **c** absorption coefficient of CMP-T, CMP-B and CMP-BG. SE_T_, SE_A_ and SE_R_ of **d** CMP-T, **e** CMP-B and **f** CMP-BG in X band. PLD distribution maps of structures with **g** CMP-T, **h** CMP-B and **i** CMP-BG. **j** Comparison of EMI SE and R with those of previously reported EMI shielding materials. **k** EMI shielding mechanism of CMP

The EMI shielding performance of MXene/PI aerogels with identical thickness but different structures (vertical, disordered, and horizontal) was systematically investigated, and the thicknesses of the different structures are presented in Table S1 and Fig. S11. The results indicate that the horizontally structured MP exhibits the highest total EMI SE, albeit with a relatively high reflection coefficient (R). In contrast, the vertically structured MP demonstrates the lowest reflection but shows a diminished SE_T_. The disordered MP displays intermediate values for both SE_T_ and R, situating its performance between the horizontal and vertical configurations (Fig. S12). Carbonized vertical, disordered, and horizontal C-MXene/PI aerogels exhibit consistent structural-property correlations, rigorously adhering to the aforementioned EMI shielding mechanism (Fig. S13). A previous study developed an asymmetric dual-layer MXene/graphene oxide aerogel, achieving a synergistic mechanism of absorption–reflection-reabsorption, thereby achieving broadband, absorption-dominated electromagnetic interference shielding effect [23]. This study constructed and precisely controlled a three-layer (vertical-disordered-horizontal) heterogeneous structure. The results showed that the total EMI shielding performance of the heterolayered aerogel was far superior to that of the vertical structure and disordered structure aerogels, and was comparable to that of the horizontal structure aerogel (Figs. 2a and S14). This similarity may be attributed to the high-loss horizontal layered structure, which eliminates the need for an excessively thick shielding layer. The EMI shielding performance of CMP with hierarchical anisotropic structure is investigated with differing electromagnetic wave incidence angles. The average SE_T_ and SE_R_ in X-band were 91.4 and 6.0 dB for the aerogel with vertical-disordered-horizontal (CMP-T) pore orientation, while that were 91.3 and 2.4 dB for the aerogel with horizontal-disordered-vertical (CMP-B) pore orientation (Fig. 2b, d, e and Table S2). This finding indicates that while the total shielding effectiveness remains comparable for electromagnetic waves incident from different directions, waves penetrating through the vertically structured surface (aligned with the electromagnetic wave propagation direction) exhibit enhanced penetration depth, facilitating more effective internal dissipation within the aerogel. To further enhance the absorption coefficient of C-MXene/PI aerogel, a gradient conductive structure layer ranging from vertically aligned to disordered to horizontally aligned was constructed. This architecture maintains constant PAS content while increasing the MXene concentration along the structural gradient. The electrical conductivity progressively increases from the vertical layer to the disordered layer and then to the horizontal layer (Fig. S15). Figure 2c shows the gradient conductive-structured CMP-BG achieves low reflection coefficient of 0.40, surpassing that of its non-gradient counterpart CMP-B (R = 0.42). Furthermore, CMP-BG maintains exceptional EMI shielding effectiveness (91.0 dB) within the X-band frequency range (Fig. 2f). Furthermore, we investigated the performance stability of the C-MXene/PI aerogel under damp-heat conditions. The results indicate that after exposure to an environment of 90 °C and 80%-90% relative humidity for ten days, the EMI shielding performance remained largely unchanged. XRD analysis confirmed that the phase composition of the C-MXene/PI aerogel was essentially preserved after damp-heat exposure, consisting primarily of amorphous carbon and TiC (Fig. S16). Therefore, the C-MXene/PI aerogel demonstrates both high EMI shielding effectiveness and a low reflection coefficient, showcasing promising potential for applications in harsh damp-heat environments.

To further understand the transmission behavior of incident EMW within CMP aerogels, the power loss density (PLD) distribution, electric field (EF) distribution and electrical energy density (EED) distribution of CMP-T, CMP-B, and CMP-BG aerogels at 10 GHz were simulated through finite element simulations (Figs. 2g–i and S18–S19). The simulated PLD distribution patterns demonstrate that electromagnetic dissipation in CMP-T primarily localizes within the basal horizontal structural layer, with limited penetration depth of electromagnetic waves into the aerogel interior, thus resulting in a relatively elevated reflection coefficient. Significantly, when electromagnetic waves are incident from the vertically structured surface, the PLD profiles indicate global energy dissipation throughout the aerogel matrix. Notably, the progressively graded conductive architecture of CMP-BG induces enhanced localized energy dissipation, particularly within the intermediate disordered phase and the underlying horizontal lamellar structure, as evidenced by intensified PLD concentrations. The EF and EED distribution maps corroborate these observations, revealing synergistic electromagnetic attenuation mechanisms involving collective contributions of impedance matching optimization and multi-scale polarization effects. The research progress of various EMI shielding materials in other reported literature was summarized, and the results are shown in Figs. 2j, S20, and S21 [24–32]; Tables S3 and S4. The heterolayered CMP aerogel with electrically conductive gradient structure exhibited high normalized EMI SE and ultra-low reflection coefficient of 0.40, which is better than previously reported. In addition, benefiting from the low density (12 mg cm^−3^) and high EMI shielding effectiveness (91.0 dB) of CMP-BG, the SSE (SE_T_/density × thickness) is up to 7583.3 dB cm^2^ g^−1^.

Based on the aforementioned results, a schematic of the absorption mechanism of the multilayer aerogels is shown in Fig. 2k. On the macroscale, the hierarchical anisotropic structure of the aerogel synergistically optimizes the overall impedance matching and enhances internal energy dissipation. Specifically, the vertically aligned channels, whose elongation direction parallels the wave propagation direction, generate numerous air-material interfaces, thereby improving impedance matching and promoting wave penetration over surface reflection. The disordered structure, with its random and isotropic porous network, induces internal reflections and multiple scattering of electromagnetic waves. The horizontal architecture creates extensive continuous conductive interfaces perpendicular to the wave propagation direction, through which electromagnetic waves are significantly attenuated via multi-layer reflections and conductive loss between adjacent conductive sheets. This layered anisotropic structure enables electromagnetic waves to undergo a stepwise absorption-dissipation-re-dissipation process. The dielectric parameter gradients arising from filler concentration variations between adjacent layers in the progressively graded conductive structure induce significant interfacial impedance mismatches, which further amplify interlayer multiple reflections. Concurrently, the MXene/PI-derived interfacial heterojunctions and defect-induced dipolar polarization synergistically contribute to electromagnetic energy absorption. Collectively, the excellent EMI shielding effectiveness and low reflection coefficient of this layered anisotropic CMP aerogel are attributed to its multiscale anisotropic structural design. In particular, the gradual impedance changes achieved through the combination of hierarchical conductive heterostructures, and the synergistic effects generated by enhanced energy dissipation (through interfaces and dipole polarization) facilitated by heterointerfaces and defect states play an important role in its excellent shielding properties.

### THz EMI Shielding Performances of the C-MXene/PI Aerogel

**Fig. 3:**
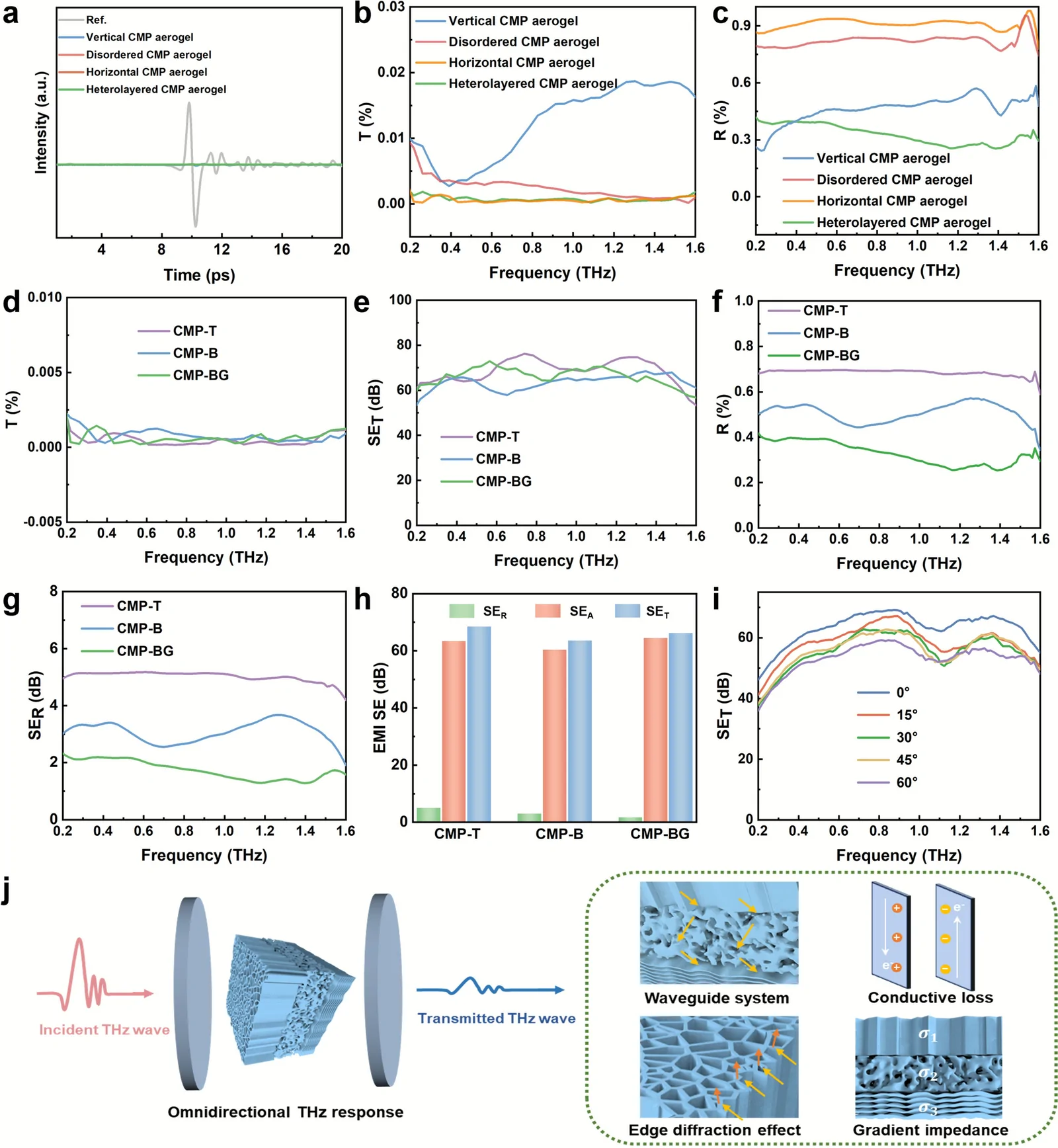
THz wave EMI shielding performance of the C-MXene/PI aerogels. **a** THz time-domain spectroscopy, **b** transmittance and **c** reflection of different structural CMP aerogels in a frequency range of 0.2–1.6 THz. **d** Transmittance, **e** SE_T_, **f** reflection and **g** SE_R_ of CMP-T, CMP-B and CMP-BG in THz. **h** SE_T_, SE_A_ and SE_R_ average value of CMP-T, CMP-B and CMP-BG. **i** Omnidirectional SE_T_ of CMP-BG aerogels with incident angle changing from 0° to 60°. **j** Schematic illustration of the omnidirectional terahertz response mechanism for heterolayered CMP aerogels

Terahertz (THz) technology, as a core component of 6G communications and military detection systems, demands the development of high-efficiency EMI shielding materials due to its high-frequency and high-energy characteristics, which are vital for ensuring communication security and fulfilling multi-scenario protection needs [33, 34] The EMI shielding performance (transmission and reflection) of CMP in the THz band was measured using terahertz time-domain spectroscopy (THz-TDS), with the schematic diagram of the testing principle illustrated in Fig. S22. Compared with the air reference, the terahertz signal showed significant attenuation during its transmission through the C-MXene/PI aerogel (Figs. 3a and S23). The lower the transmittance, the better the shielding performance of the material against terahertz waves. Experimental results show that heterolayered and horizontal-structured CMP aerogels exhibit the lowest transmittance in the 0.2–1.6 THz band (Fig. 3b), and calculated EMI SE_T_ values follow the order: horizontal CMP aerogel > heterolayered CMP aerogel > disordered CMP aerogel > vertical CMP aerogel (Fig. S24). However, compared with the horizontal and disordered structure, the reflectivity of the vertical and heterolayered aerogels is significantly lower (Figs. 3c and S25). The results indicate that, similar to the trends observed in the microwave band, heterolayered C-MXene/PI aerogels in the terahertz band combine the advantages of the three structures, achieving excellent EMI shielding performance and ultra-low reflectivity.

The variations in terahertz EMI shielding performance were also tested under electromagnetic wave incidence from different directions (CMP-T and CMP-B) and with the introduction of a progressive conductivity gradient (CMP-BG). The average transmittance of CMP-T, CMP-B, and CMP-BG in the 0.2–1.6 THz range was 4.4 × 10^–7^–1.4 × 10^–7^ (Fig. 3d), and their average EMI SE_T_ values ranged from 63.6 to 68.5 dB (Fig. 3e). The results indicate that the incident direction of electromagnetic waves and the progressive allocation of conductivity gradients have minimal impact on the overall EMI shielding performance. However, as clearly shown in Fig. 3f, g, CMP-BG with a progressive concentration gradient and vertical structural incidence exhibits the lowest reflection coefficient (R = 0.33) and reflection value (SE_R_ = 1.7). Furthermore, the SE_T_ values of CMP-T, CMP-B and CMP-BG aerogels are 68.5, 63.6, and 66.2 dB respectively, where it is observed that the SE_A_ values have significantly increased while the SE_R_ values have significantly decreased (Fig. 3h). This result reveals that the introduction of structural design and the gradual increase in gradient degree have a positive effect on enhancing terahertz absorption. The impact of varying incident angles from 15° to 60° on the EMI shielding performance of CMP-BG was tested. The results demonstrate that the CMP-BG aerogel is insensitive to the incident angle of terahertz waves, which can be attributed to the wave-buffering effect of its heterolayered structure. The gradient porous structure of the outer layer serves as an impedance-matching zone, enabling efficient wave penetration rather than surface reflection across the entire angular spectrum. Once electromagnetic waves enter the aerogel, the intermediate disordered layer with randomly distributed interfaces and conductive networks induces intensive multiple scattering and internal reflections. The structurally isotropic nature of this layer makes its dissipation mechanism largely independent of the wave incidence direction. Finally, the remaining wave energy is further dissipated by the inner horizontal layer. Through this coordinated multi-stage dissipation mechanism, the heterostructured CMP-BG aerogel achieves stable and highly efficient shielding performance over a wide angular range, as conclusively demonstrated in Fig. 3i. Figure 3j demonstrates the multi-directional shielding of terahertz electromagnetic waves by CMP and the EMI shielding mechanism. The abundant pores in the aerogel generate additional secondary reflections at large incidence angles by distorting the electromagnetic fields on its surface, thereby inducing multiple oscillations and diffraction effects. The highly conductive pore walls act as a guiding system for electromagnetic waves, dissipating them through conduction loss. Furthermore, the tri-heterolayered structure reduces the dependence on incidence angles and local modes relative to tubular configurations along the radial direction and periodic pore-structured tubular configurations. The progressive conductive gradient architecture minimizes impedance step differences between each layer to ensure terahertz waves penetrate the aerogel interior without significant reflection. In summary, the heterolayered CMP aerogel with electrically conductive gradient structured achieves highly efficient EMI shielding across both microwave and terahertz bands, while maintaining lightweight characteristics and low-reflection properties.

### Infrared Stealth Performance of C-MXene/PI Aerogel

**Fig. 4:**
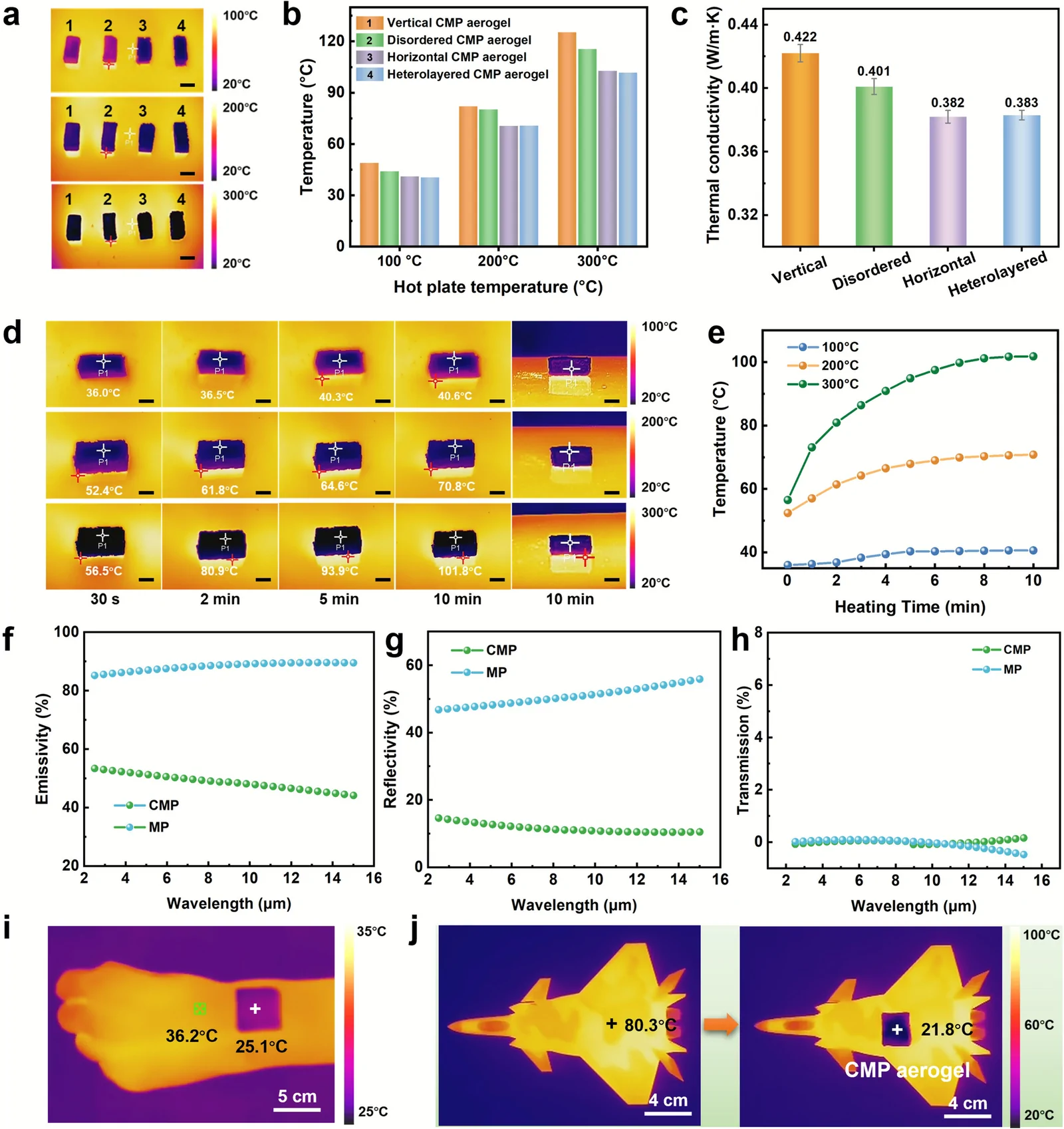
Infrared Stealth Performance of C-MXene/PI aerogel. **a** Infrared thermal images, **b** surface temperature and **c** thermal conductivity of different structured CMP. The scale bar is 1.0 cm in infrared thermal images. **d** Infrared thermal images and **e** the surface temperature variations of heterolayered CMP during the heating process. The scale bar is 1.0 cm in infrared thermal images. **f** Infrared emissivity, **g** reflectivity and **h** transmission of MP and CMP in the 2–16 μm. **i** CMP aerogel placed on the arm for infrared thermal imaging. **j** Thermal infrared images of stealth fighter model covered with aerogel under 80 °C

Modern military detection systems have rendered single-band stealth materials insufficient for protecting military assets. A critical need exists for stealth materials capable of simultaneously suppressing thermal infrared radiation and microwave/terahertz reflection [35, 36]. To investigate the thermal transport properties of aerogels with different architectures, C-MXene/PI aerogel featuring vertical, disordered, horizontal, and Heterolayered structures were placed on the heating stage, as depicted in Fig. S26. The results demonstrate that on the high-temperature stage, the vertically oriented aerogel exhibited the highest surface radiation temperature, whereas the heterolayered aerogel exhibited the lowest (Fig. 4a, b). The thermal conductivity of four different structured CMP were tested. The horizontal structure and heterolayered CMP exhibited lower thermal conductivities, which were 0.382 and 0.383 W m^−1^ K^−1^ respectively (Fig. 4c). This is attributed to the horizontal structure aerogel effectively blocks heat conduction, while the the heterolayered C-MXene/PI aerogel has an even more extreme distortion on solid heat conduction, effectively restricting the movement of gas molecules [37].

To further evaluate infrared stealth performance, heterolayered C-MXene/PI aerogel were placed on heating stages at 100, 200, and 300 °C to investigate changes in the aerogel surface radiation temperature (Fig. 4d). The surface radiation temperature gradually increased and stabilized after approximately 8 min. At the 10 min mark, the surface radiation temperatures were 40.6, 70.8, and 101.8 °C, respectively. The underlying mechanism originates from two critical factors: Firstly, the high porosity and ultralow density of C-MXene/PI aerogels governs surface temperature reduction through elongating phononn transport pathways, thereby enhancing the overall thermal resistance. Secondly, low infrared emissivity is another key factor contributing to superior infrared stealth performance. The infrared reflectance, absorbance, and transmittance of the MP and CMP aerogels across the 2–16 μm wavelength range are shown in Fig. 4f–h. The increased graphitization degree resulting from carbonization endows the MXene/PI aerogel with an extremely low infrared emissivity [38]. The C-MXene/PI aerogel achieved minimum average emissivities of 52.1% and 47.9% in the 3–5 and 8–12 μm bands, respectively. In the 2–15 μm wavelength range, the emissivity values measure 53.1% for the vertically aligned structure, 48.4% for the disordered structure, and 46.8% for the horizontally aligned structure (Fig. S27), showing relatively minor differences compared to the hierarchical anisotropic C-MXene/PI aerogel. These results demonstrate that the excellent infrared stealth performance of the C-MXene/PI aerogel is achieved through the synergistic combination of low thermal conductivity and moderate emissivity. While the low emissivity helps reduce radiative heat exchange, the ultra-low thermal conductivity of 0.383 W m^−1^ K^−1^ plays a dominant role by fundamentally minimizing heat transfer from the protected object to the aerogel surface. This effectively lowers the surface temperature to nearly match the ambient environment, significantly reducing the thermal contrast detectable by infrared systems. When placed on a human arm, the CMP aerogel exhibits the same color as the background, demonstrating excellent thermal camouflage performance (Fig. 4i). As shown in Fig. 4i, the CMP aerogel was positioned on an aircraft fighter model heated to 80 °C, simulating an operational, heat-emitting fighter. The surface radiative temperature of the CMP aerogel reached 21.8 °C, approaching the ambient temperature of 20 °C (Fig. 4j), effectively achieving infrared stealth for the fighter model. These findings indicate that the heterolayered CMP aerogel can effectively prevent detection by external infrared surveillance of both operational heat-emitting fighter aircraft and personnel. In summary, the C-MXene/PI aerogel exhibits exceptional infrared stealth characteristics, demonstrating promising application potential in high-tech fields such as aerospace, stealth weaponry, and electromagnetic protection.

## Conclusion

In summary, a heterolayered C-MXene/PI aerogel featuring spatially programmed electrical conductivity gradients, fabricated via a stepwise freezing-driven assembly process followed by freeze-drying is presented. The C-MXene/PI aerogel exhibits excellent EMI shielding performance in the microwave and terahertz frequency bands. The average EMI SE_T_ values for the X-band and the 0.2–1.6 THz band are 91.0 and 66.2 dB, respectively. Benefitting from the layered structure and the introduction of conductivity gradients, the C-MXene/PI aerogel demonstrates an extremely low reflection coefficient (R = 0.40 in the X-band and R = 0.33 in the THz band). Additionally, the C-MXene/PI aerogel possesses a low thermal conductivity of 0.383 W m^−1^ K^−1^ and exhibits an extremely low infrared emissivity, demonstrating potential applications in infrared stealth and thermal camouflage. This work presents an effective strategy for fabricating materials with low reflection and high effectiveness, achieving multi-spectrum compatibility across microwave, terahertz, and infrared regions.

## Supplementary Information

Below is the link to the electronic supplementary material.

Supplementary file1 (DOCX 11835 KB)

## References

[1] Y. Zhao, S. Tan, J. Yu, R. Yu, T. Xu et al., A rapidly assembled and camouflage-monitoring-protection integrated modular unit. Adv. Mater. **37**(6), e2412845 (2025). 10.1002/adma.202412845 39690802 10.1002/adma.202412845

[2] P.-Y. Zhao, H.-L. Peng, B. Cai, L. Zhou, C.-M. Liang et al., Mechanism decoupling of impedance matching and attenuation enhancement via spatial distribution of loading components. Adv. Funct. Mater. (2025). 10.1002/adfm.202518479

[3] X. Yang, L. Liang, C. Li, B. Zhang, Y. Zhao et al., Fluid-actuated nano-micro-macro structure morphing enables smart multispectrum compatible stealth. Nano Lett. **25**(1), 569–577 (2025). 10.1021/acs.nanolett.4c05494 39698853 10.1021/acs.nanolett.4c05494

[4] X. Feng, C. Li, J. Song, Y. He, W. Qu et al., Differential perovskite hemispherical photodetector for intelligent imaging and location tracking. Nat. Commun. **15**(1), 577 (2024). 10.1038/s41467-024-44857-4 38233400 10.1038/s41467-024-44857-4 PMC10794423

[5] H.J. Kwon, J.-H. Park, S.J. Suh, Multilayered Cu/NiFe thin films for electromagnetic interference shielding at high frequency. J. Alloys Compd. **914**, 165330 (2022). 10.1016/j.jallcom.2022.165330

[6] C. Li, L. Liang, B. Zhang, Y. Yang, G. Ji, Magneto-dielectric synergy and multiscale hierarchical structure design enable flexible multipurpose microwave absorption and infrared stealth compatibility. Nano-Micro Lett. **17**(1), 40 (2024). 10.1007/s40820-024-01549-4 10.1007/s40820-024-01549-4 PMC11480309 39407045

[7] Z. Wei, Y. Cai, Y. Zhan, Y. Meng, N. Pan et al., Ultra-low loading of ultra-small Fe_3_O_4_ nanoparticles on nonmodified CNTs to improve green EMI shielding capability of rubber composites. Small **20**(9), 2307148 (2024). 10.1002/smll.202307148 10.1002/smll.202307148 37840441

[8] Y. Cao, Z. Cheng, R. Wang, X. Liu, T. Zhang et al., Multifunctional graphene/carbon fiber aerogels toward compatible electromagnetic wave absorption and shielding in gigahertz and terahertz bands with optimized radar cross section. Carbon **199**, 333–346 (2022). 10.1016/j.carbon.2022.07.077

[9] H. Peng, B. Cai, Y. Zhang, L. Gao, P.-Y. Zhao et al., Radar-terahertz-infrared compatible stealth coaxial silver Nanowire@Carbon nano-cable aerogel. Angew. Chem. Int. Ed. **64**(10), e202421090 (2025). 10.1002/anie.202421090 10.1002/anie.202421090 39757990

[10] H.-Y. Wang, P. Hu, X.-B. Sun, Z.-L. Hou, P.-Y. Zhao et al., Bioinspired disordered aerogel for omnidirectional terahertz response. Adv. Mater. **37**(10), e2418889 (2025). 10.1002/adma.202418889 39887578 10.1002/adma.202418889

[11] A. Liu, H. Qiu, X. Lu, H. Guo, J. Hu et al., Asymmetric structural MXene/PBO aerogels for high-performance electromagnetic interference shielding with ultra-low reflection. Adv. Mater. **37**(5), 2414085 (2025). 10.1002/adma.202414085 10.1002/adma.202414085 39629529

[12] S. Habibpour, Y. Rahimi-Darestani, M. Salari, K. Zarshenas, S.M. Taromsari et al., Synergistic layered design of aerogel nanocomposite of graphene nanoribbon/MXene with tunable absorption dominated electromagnetic interference shielding. Small **20**(45), 2404876 (2024). 10.1002/smll.202404876 10.1002/smll.202404876 39072882

[13] W. Zheng, H. Xie, J. Li, H. Yu, Z. Tang et al., Design of polyimide/carbon nanotube@Ag@polyimide/graphene composite aerogel for infrared stealth and electromagnetic interference protection. Compos. Part A Appl. Sci. Manuf. **186**, 108371 (2024). 10.1016/j.compositesa.2024.108371

[14] H. Wang, Y. Jiang, Z. Ma, Y. Shi, Y. Zhu et al., Hyperelastic, robust, fire-safe multifunctional MXene aerogels with unprecedented electromagnetic interference shielding efficiency. Adv. Funct. Mater. **33**(49), 2306884 (2023). 10.1002/adfm.202306884

[15] H. Liu, Y. Shao, Z. Wang, L. Jiang, B. Mou et al., Mechanically robust and multifunctional Ti_3_C_2_T_*x*_ MXene composite aerogel for broadband EMI shielding. Carbon **221**, 118948 (2024). 10.1016/j.carbon.2024.118948

[16] Z. Wang, L. Ma, H. Ma, M. Xu, X. Wu et al., Double-layer electromagnetic shielding materials with microcellular structure for strong absorption and low reflection. J. Mater. Sci. Technol. **245**, 227–237 (2026). 10.1016/j.jmst.2025.04.056

[17] A.A. Isari, A. Ghaffarkhah, S.A. Hashemi, S. Wuttke, M. Arjmand, Structural design for EMI shielding: from underlying mechanisms to common pitfalls. Adv. Mater. **36**(24), 2310683 (2024). 10.1002/adma.202310683 10.1002/adma.202310683 38467559

[18] T. Xue, Y. Yang, D. Yu, Q. Wali, Z. Wang et al., 3D printed integrated gradient-conductive MXene/CNT/polyimide aerogel frames for electromagnetic interference shielding with ultra-low reflection. Nano-Micro Lett. **15**(1), 45 (2023). 10.1007/s40820-023-01017-5 10.1007/s40820-023-01017-5 PMC9908813 36752927

[19] T. Shi, Z. Zheng, H. Liu, D. Wu, X. Wang, Configuration of multifunctional polyimide/graphene/Fe_3_O_4_ hybrid aerogel-based phase-change composite films for electromagnetic and infrared bi-stealth. Nanomaterials **11**(11), 3038 (2021). 10.3390/nano11113038 34835800 10.3390/nano11113038 PMC8620502

[20] T. Shi, Z. Zheng, H. Liu, D. Wu, X. Wang, Flexible and foldable composite films based on polyimide/phosphorene hybrid aerogel and phase change material for infrared stealth and thermal camouflage. Compos. Sci. Technol. **217**, 109127 (2022). 10.1016/j.compscitech.2021.109127

[21] J. Jing, H. Liu, X. Wang, Long-term infrared stealth by sandwich-like phase-change composites at elevated temperatures *via* synergistic emissivity and thermal regulation. Adv. Funct. Mater. **34**(2), 2309269 (2024). 10.1002/adfm.202309269

[22] T. Shi, J. Jing, Z. Qian, G. Wu, G. Tian et al., Sandwich-structured fluorinated polyimide aerogel/paraffin phase-change composites simultaneously enables gradient thermal protection and electromagnetic wave transmission. Adv. Sci. **12**(5), 2411758 (2025). 10.1002/advs.202411758 10.1002/advs.202411758 PMC11791978 39639801

[23] C.-Z. Qi, P. Min, X. Zhou, M. Jin, X. Sun et al., Multifunctional asymmetric bilayer aerogels for highly efficient electromagnetic interference shielding with ultrahigh electromagnetic wave absorption. Nano-Micro Lett. **17**(1), 291 (2025). 10.1007/s40820-025-01800-6 10.1007/s40820-025-01800-6 PMC12162446 40504423

[24] T. Mai, L. Chen, Q. Liu, Z.-H. Guo, M.-G. Ma, Zeolitic imidazolate frameworks derived magnetic nanocage/MXene/nanocellulose bilayer aerogels for low reflection electromagnetic interference shielding and light-to-heat conversion. Adv. Funct. Mater. **35**(13), 2417947 (2025). 10.1002/adfm.202417947

[25] M. Ma, W. Tao, X. Liao, S. Chen, Y. Shi et al., Cellulose nanofiber/MXene/FeCo composites with gradient structure for highly absorbed electromagnetic interference shielding. Chem. Eng. J. **452**, 139471 (2023). 10.1016/j.cej.2022.139471

[26] J. Zhang, L. Zeng, X. Liu, D. Zhang, A. Gao et al., Lattice-filler dual-gradient and hierarchical porous architectures customized by multiple-nozzle 3D printing towards excellent absorption-dominant electromagnetic interference shielding. Compos. Sci. Technol. **262**, 111058 (2025). 10.1016/j.compscitech.2025.111058

[27] L. Wang, J. Men, W. Ren, Z. Yang, Y. Gao et al., Low-reflection electromagnetic interference shielding composite foams with asymmetric structure towards infrared camouflage and response switching. J. Colloid Interface Sci. **697**, 137941 (2025). 10.1016/j.jcis.2025.137941 40440758 10.1016/j.jcis.2025.137941

[28] M. Sun, Z. Wang, J. Xiao, X. Tian, X. Ma et al., AgNWs/Fe_3_O_4_@NC conductive network hierarchical assembly to prepare flexible EMI shielding textile. Small **20**(14), 2304622 (2024). 10.1002/smll.202304622 10.1002/smll.202304622 37988675

[29] W. Feng, L. Zou, C. Lan, S. E, X. Pu, Core-sheath CNT@MXene fibers toward absorption-dominated electromagnetic interference shielding fabrics. Adv. Fiber Mater. **6**(5), 1657–1668 (2024). 10.1007/s42765-024-00452-2

[30] S. Li, Y. Du, H. Ye, J. Wu, Y. Wang et al., All-natural self-bonded biocomposite providing superior electromagnetic interference shield performance with effective absorption. Adv. Funct. Mater. **34**(41), 2406282 (2024). 10.1002/adfm.202406282

[31] Y. Wang, Y. Zhong, J. Kang, B. Zhang, Z. Ma et al., Multifunctional rigid polyimide foams with outstanding EMI shielding and wave absorption *via* densification strategy. J. Mater. Sci. Technol. **227**, 155–163 (2025). 10.1016/j.jmst.2024.12.021

[32] D. Gao, S. Guo, Y. Zhou, B. Lyu, X. Li et al., Absorption-dominant, low-reflection multifunctional electromagnetic shielding material derived from hydrolysate of waste leather scraps. ACS Appl. Mater. Interfaces **14**(33), 38077–38089 (2022). 10.1021/acsami.2c10787 35971686 10.1021/acsami.2c10787

[33] S. Yang, Z. Lin, X. Wang, J. Huang, R. Yang et al., Stretchable, transparent, and ultra-broadband terahertz shielding thin films based on wrinkled MXene architectures. Nano-Micro Lett. **16**(1), 165 (2024). 10.1007/s40820-024-01365-w 10.1007/s40820-024-01365-w PMC10987438 38564038

[34] R. Su, P. Liu, J. Chen, W. Wang, X. Chen et al., Self-assembly and 3D printing of SiCw@MXene/SiOC metastructure toward simultaneously excellent terahertz electromagnetic interference (EMI) shielding and electron-to-thermal conversion properties. Adv. Funct. Mater. **35**(30), 2500970 (2025). 10.1002/adfm.202500970

[35] B.-F. Guo, Y.-J. Wang, C.-F. Cao, Z.-H. Qu, J. Song et al., Large-scale, mechanically robust, solvent-resistant, and antioxidant MXene-based composites for reliable long-term infrared stealth. Adv. Sci. **11**(17), 2309392 (2024). 10.1002/advs.202309392 10.1002/advs.202309392 PMC11077694 38403451

[36] Z. Huang, A. Tong, T. Xing, A. He, Y. Luo et al., Regenerated cellulose/lignin composite aerogel with unique toast-like structure and their potential applications in thermal camouflage. Adv. Funct. Mater. **35**(6), 2414696 (2025). 10.1002/adfm.202414696

[37] X. Yin, Y. Wang, N. Pang, C. Liu, Y. Xu et al., Ultralight, highly elastic ZrO_2_/carbon fiber reinforced reduced graphene oxide aerogels with radar and infrared stealth capabilities. Compos. Part B Eng. **303**, 112628 (2025). 10.1016/j.compositesb.2025.112628

[38] X. Tan, W. Gu, Z. Tao, X. Chen, S. Li et al., Carbon-based materials for radar-infrared compatible stealth technology. Chem. Eng. J. **507**, 160168 (2025). 10.1016/j.cej.2025.160168

