# Supplementary material for: Heterolayered Carbonized MXene/Polyimide Aerogel for Low-Reflection Electromagnetic Interference Shielding and Multi-Spectrum Compatible Protection
Source: Nanomicro Lett. 2026 Jan 13;18:204. doi: 10.1007/s40820-025-02027-1 (PMC12799823; doi:10.1007/s40820-025-02027-1)
Supplement: Supplementary file 1 — Supplementary file1 (DOCX 11835 KB) [file 40820_2025_2027_MOESM1_ESM.docx]

Supporting Information for

**Heterolayered** **Carbonized MXene/Polyimide** **Aerogel for** **Multi-Spectrum** **Compatible Protection with Low Reflection**

Shan Zhang^1,2, †^, Chen-Ming Liang^1,2, †^, Lu Zhou^1,2, †^, Juntao Wu^1,2, *^, Martin C. Koo ^1,2^, Zongxin Wu^2^, Yun-Tian Chen^1,2^ and Guang-Sheng Wang^1,2, *^

^1^ State Key Laboratory of Bioinspired Interfacial Materials Science, Bioinspired Science Innovation Center, Hangzhou International Innovation Institute, Beihang University, Hangzhou 311115, P. R. China

^2^School of Chemistry, Beihang University, Beijing 100191, P. R. China

†Shan Zhang, Chen-Ming Liang, and Lu Zhou contributed equally to this work.

*Corresponding authors. E-mail: [wjt@buaa.edu.cn](mailto:wjt@buaa.edu.cn) (Juntao Wu); [wanggsh@buaa.edu.cn](mailto:wanggsh@buaa.edu.cn) (Guang-Sheng Wang)

**Supplementary Figures and Tables**


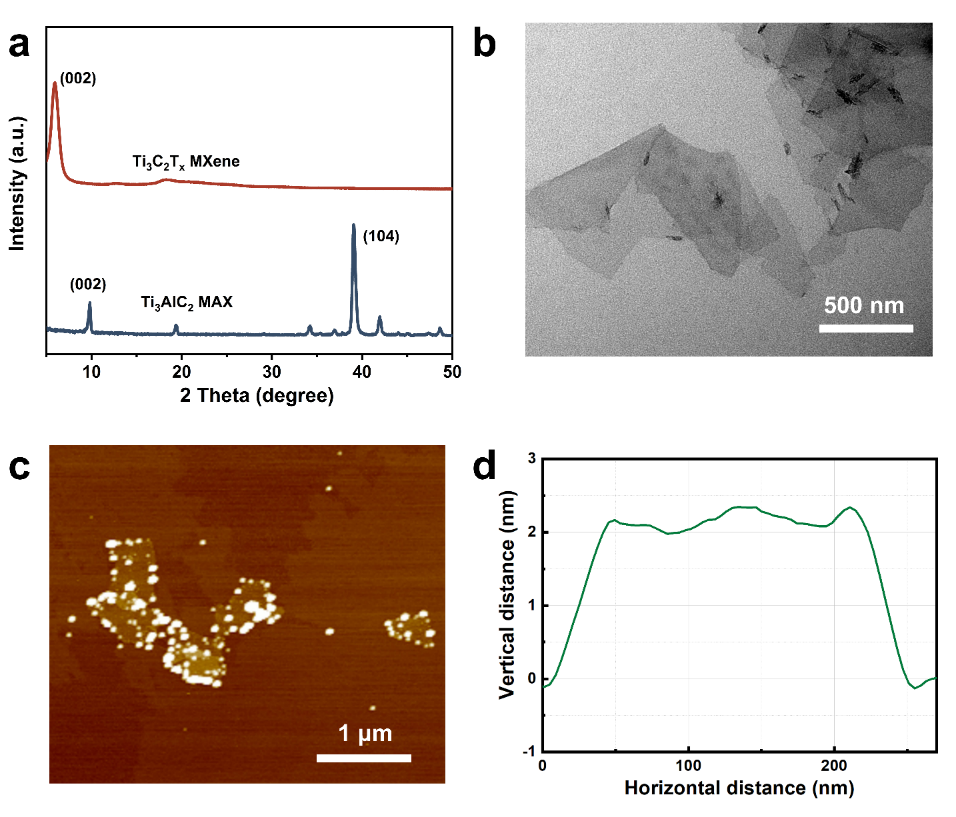


**Fig. S1** (**a**) XRD patterns of Ti_3_AlC_2_ MAX powders and Ti_3_C_2_T_x_ MXene film (**b**) TEM image of Ti_3_C_2_T_x_ MXene flakes (**c**) AFM image and (**d**) height profiles of the Ti_3_C_2_T_x_ MXene flakes


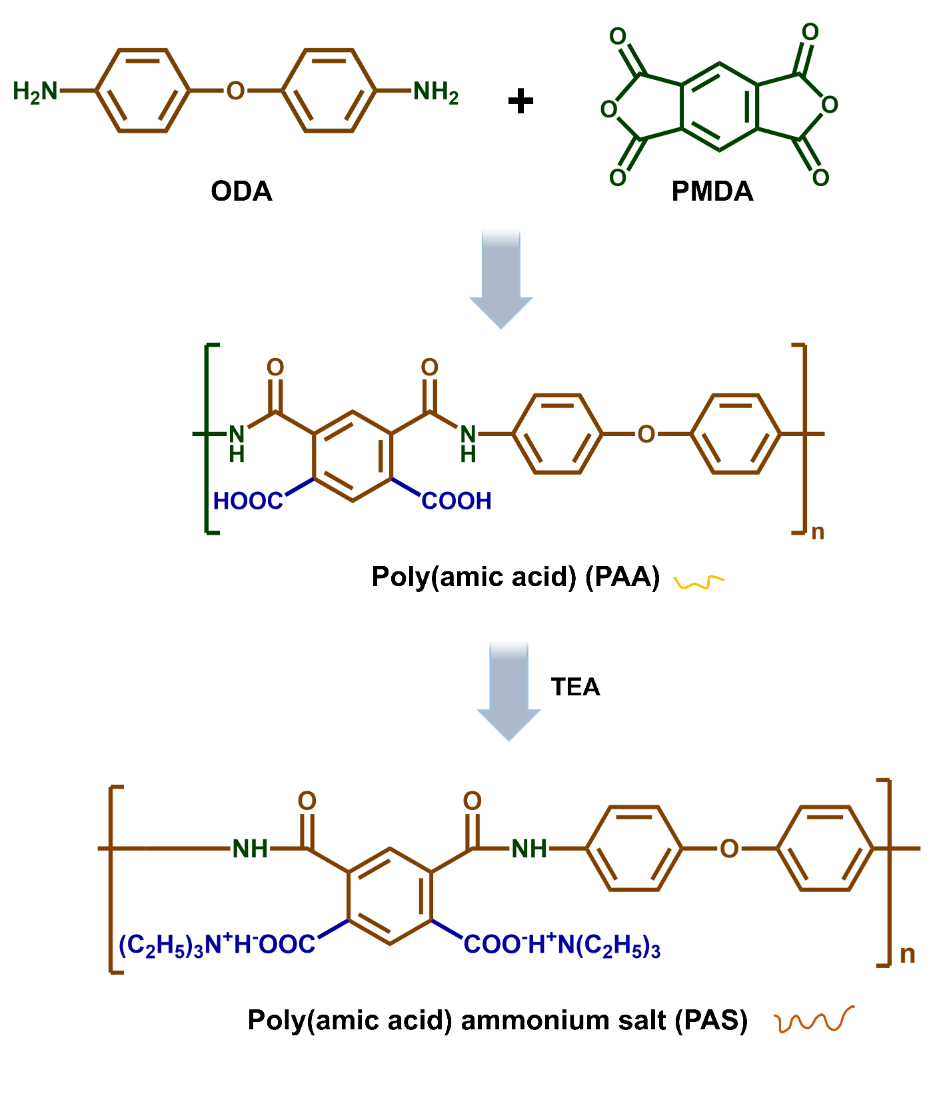


**Fig. S2** The synthesis process of poly(amic acid) (PAA) and poly(amic acid) ammonium salt (PAS)


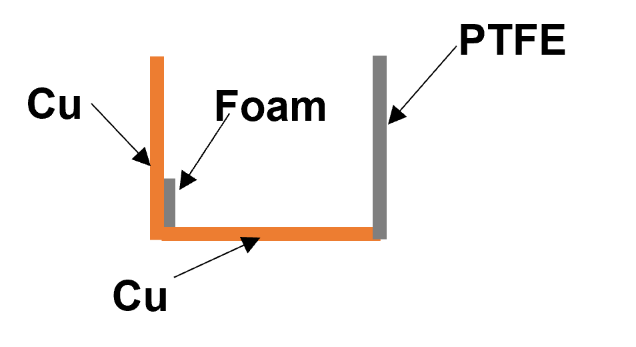


**Fig. S3** Schematic diagram of self-made freeze-drying mold

_
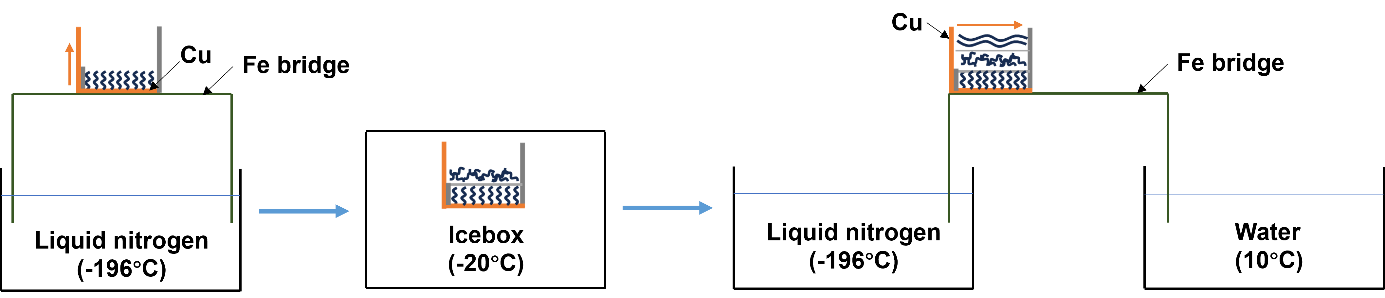
_

**Fig. S4** Schematic diagram of the process for preparing three-layer heterolayered structures by stepwise freezing


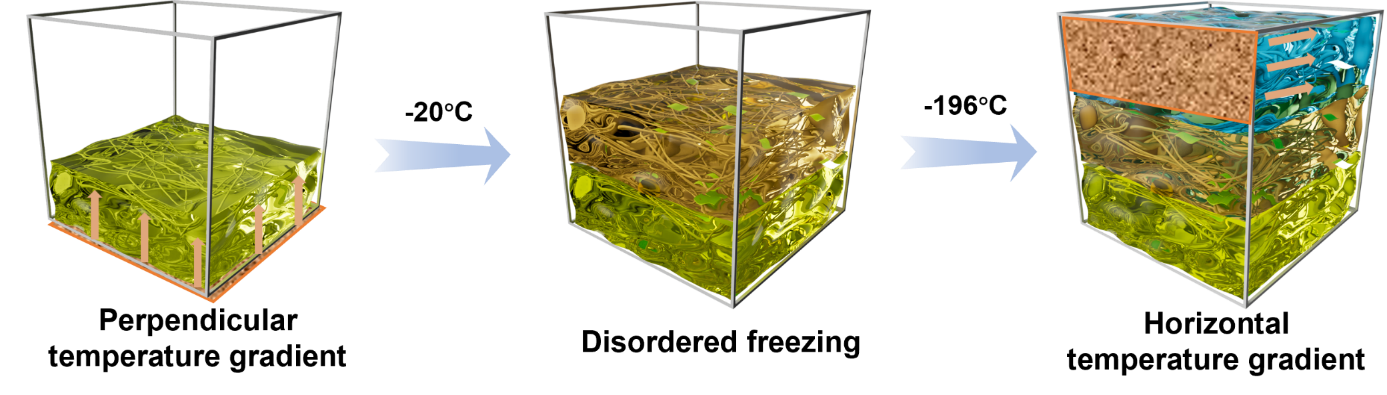


**Fig. S5** The MXene/PI aerogel was prepared by stepwise freezing. The first layer of ice crystals grew from bottom to top in liquid nitrogen. The second layer of ice crystals grew randomly in the refrigerator. The third layer of ice crystals grew horizontally along the left copper plate

**
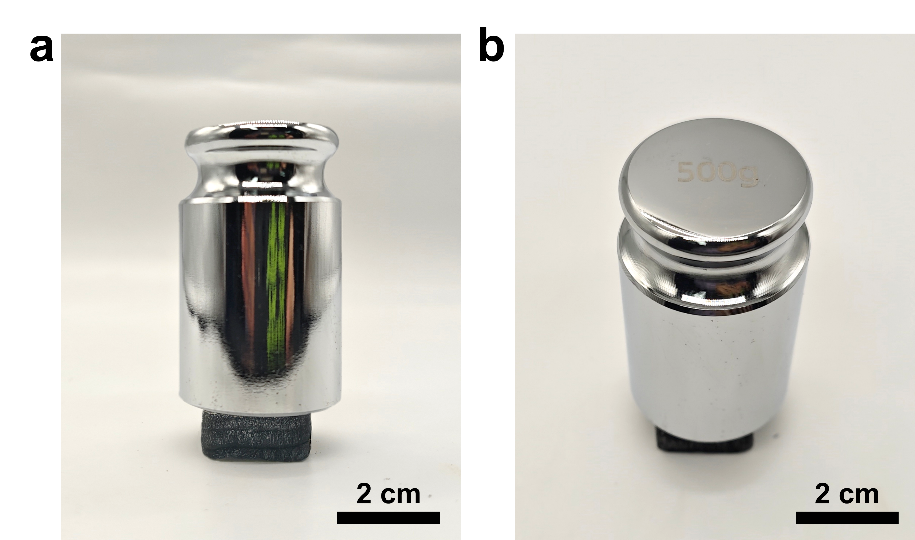
**

**Fig. S6** (**a**) Front view and (**b**) top view photos of the C-MXene/PI aerogel supporting a 500 g weight


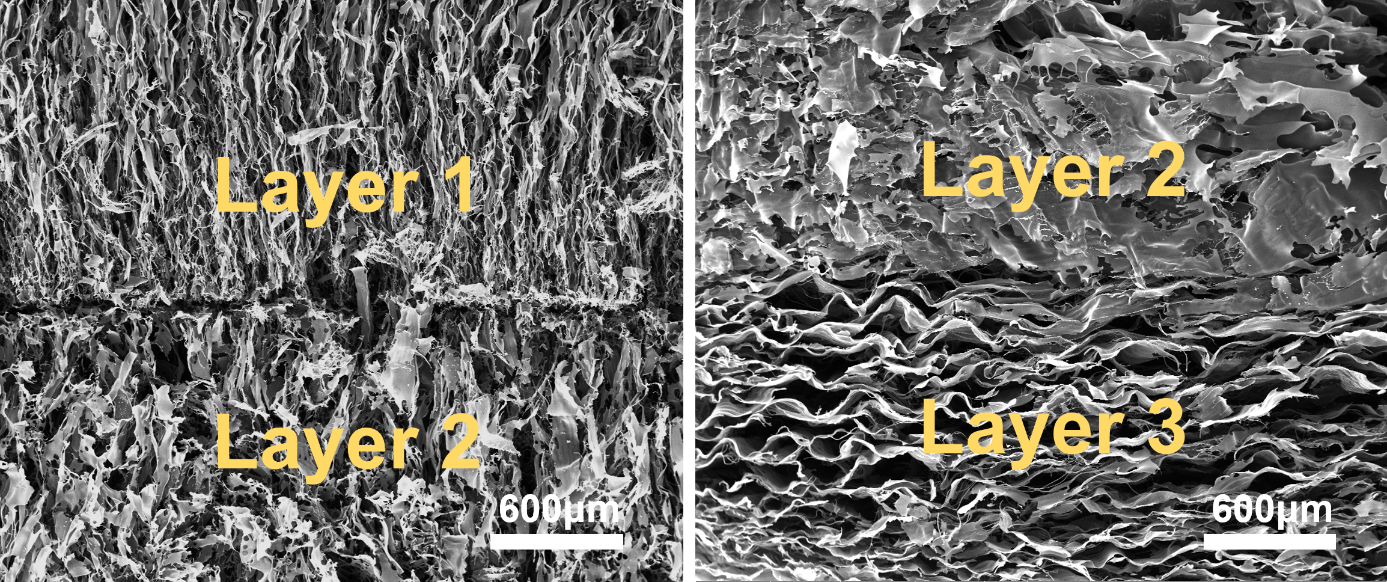


**Fig. S7** The cross-sectional SEM images of the radial sections of the three layers


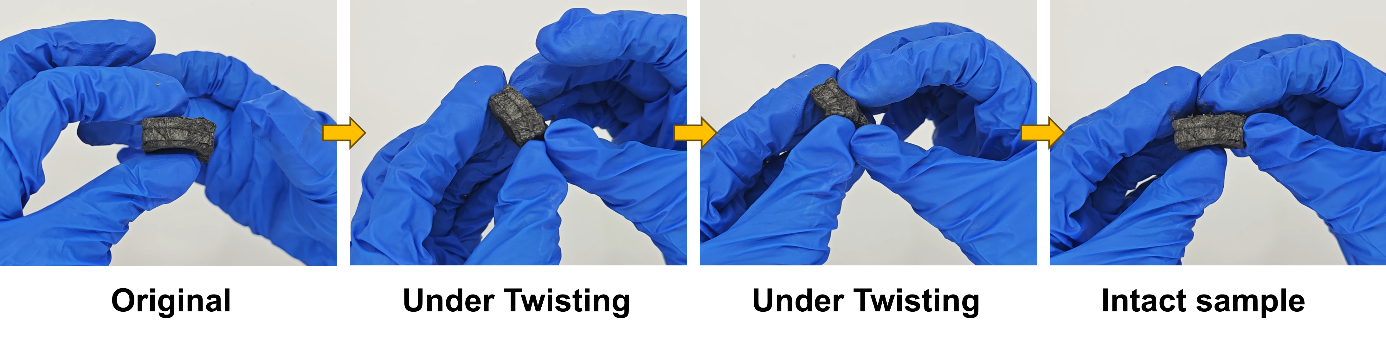


**Fig. S8** Photos of the mechanical torsion test for the C-MXene/PI aerogel


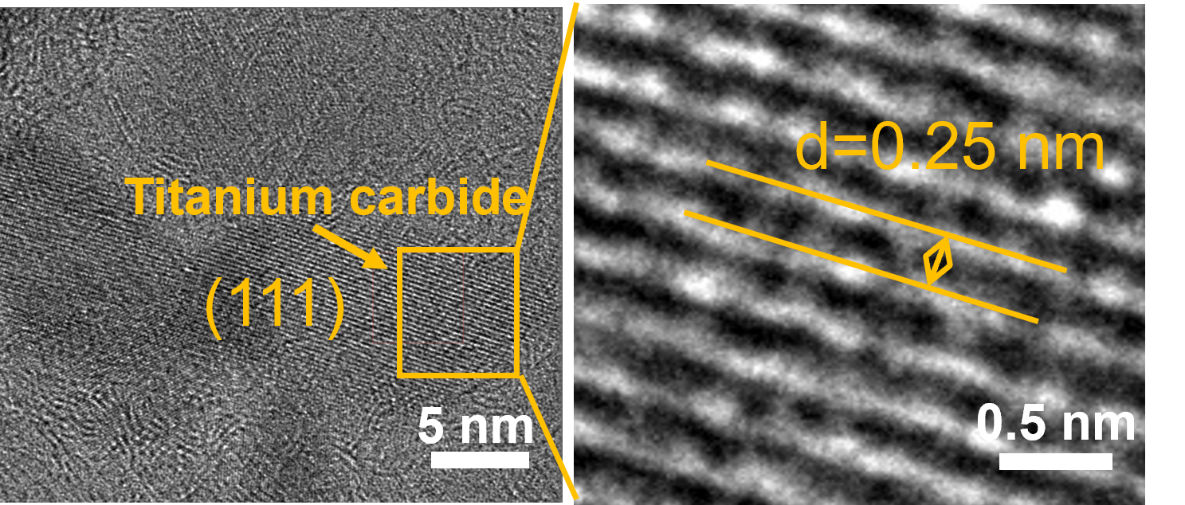


**Fig. S9** The lattice fringes of the TiC (111) plane


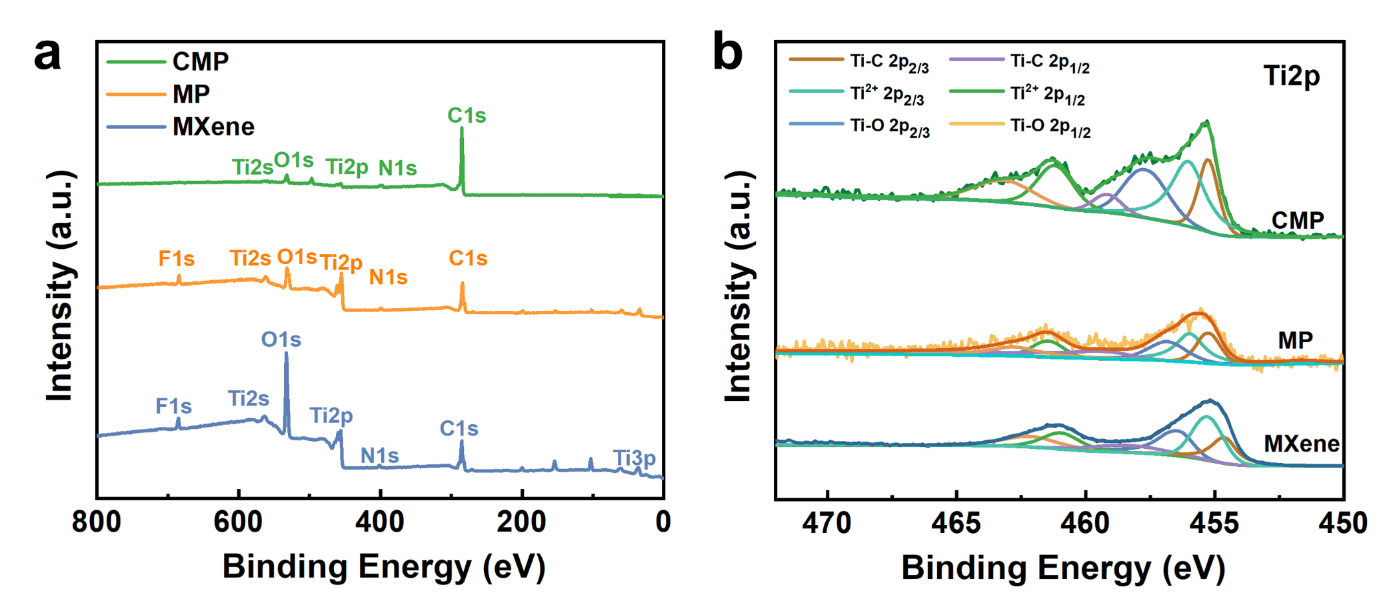


Fig. S10 (a) XPS survey spectra and (b) High-resolution XPS spectra of Ti 2p for MXene, MP and CMP


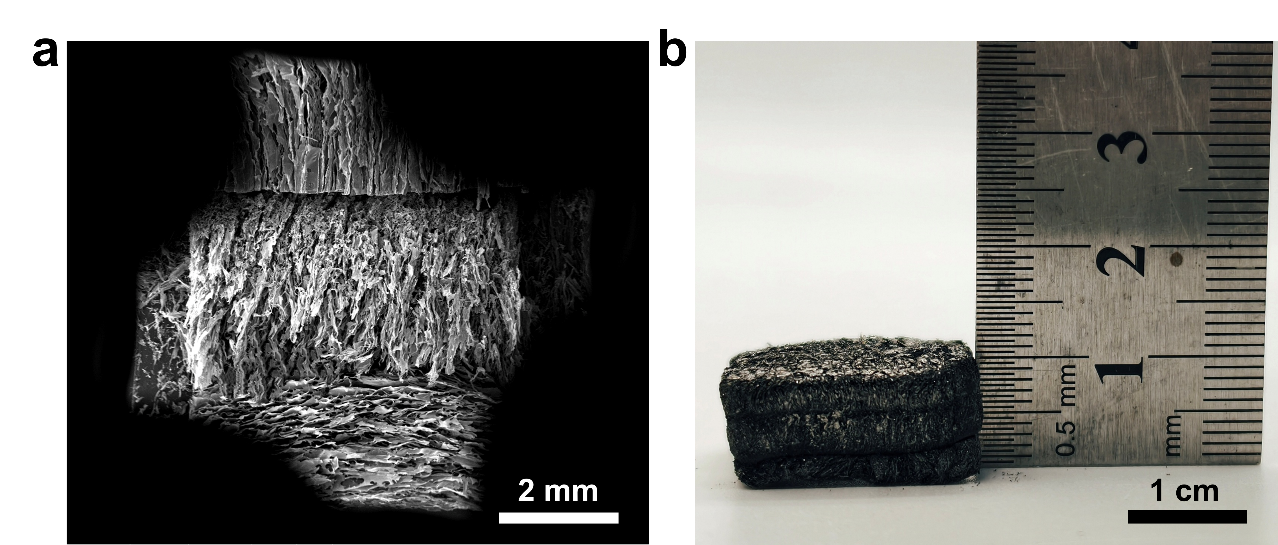


Fig. S11 (a) The cross-section SEM images of heterolayered CMP aerogel. (b) Photo of the heterolayered CMP aerogel measured with a ruler


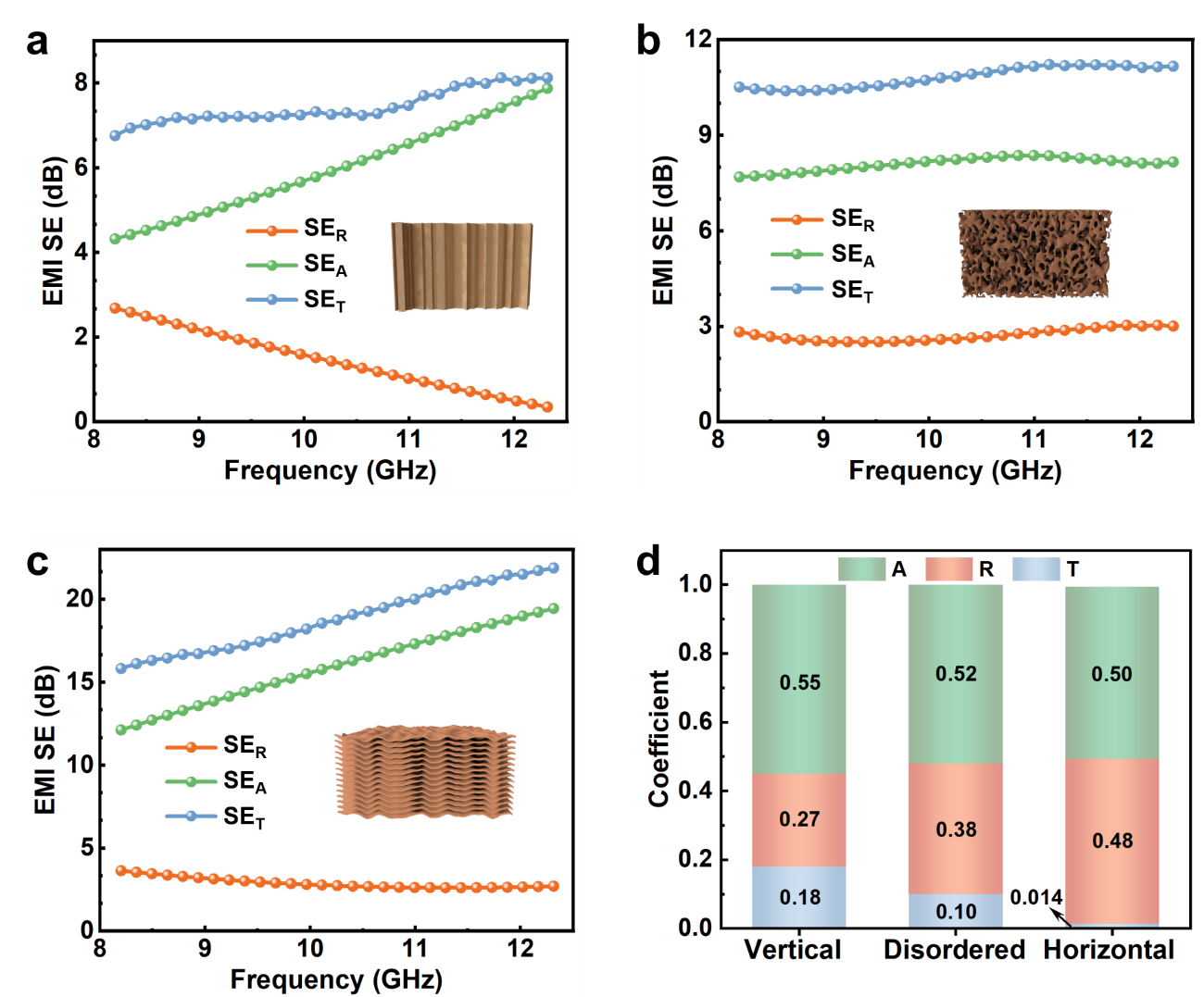


Fig. S12 EMI SE_T_, SE_R_, and SE_A_ of the (a) vertical structure MXene/PI aerogel, (b) disordered structure MXene/PI aerogel and (c) horizontal structure MXene/PI aerogel. (d) Coefficients of vertical, disordered and horizontal structure MXene/PI aerogel


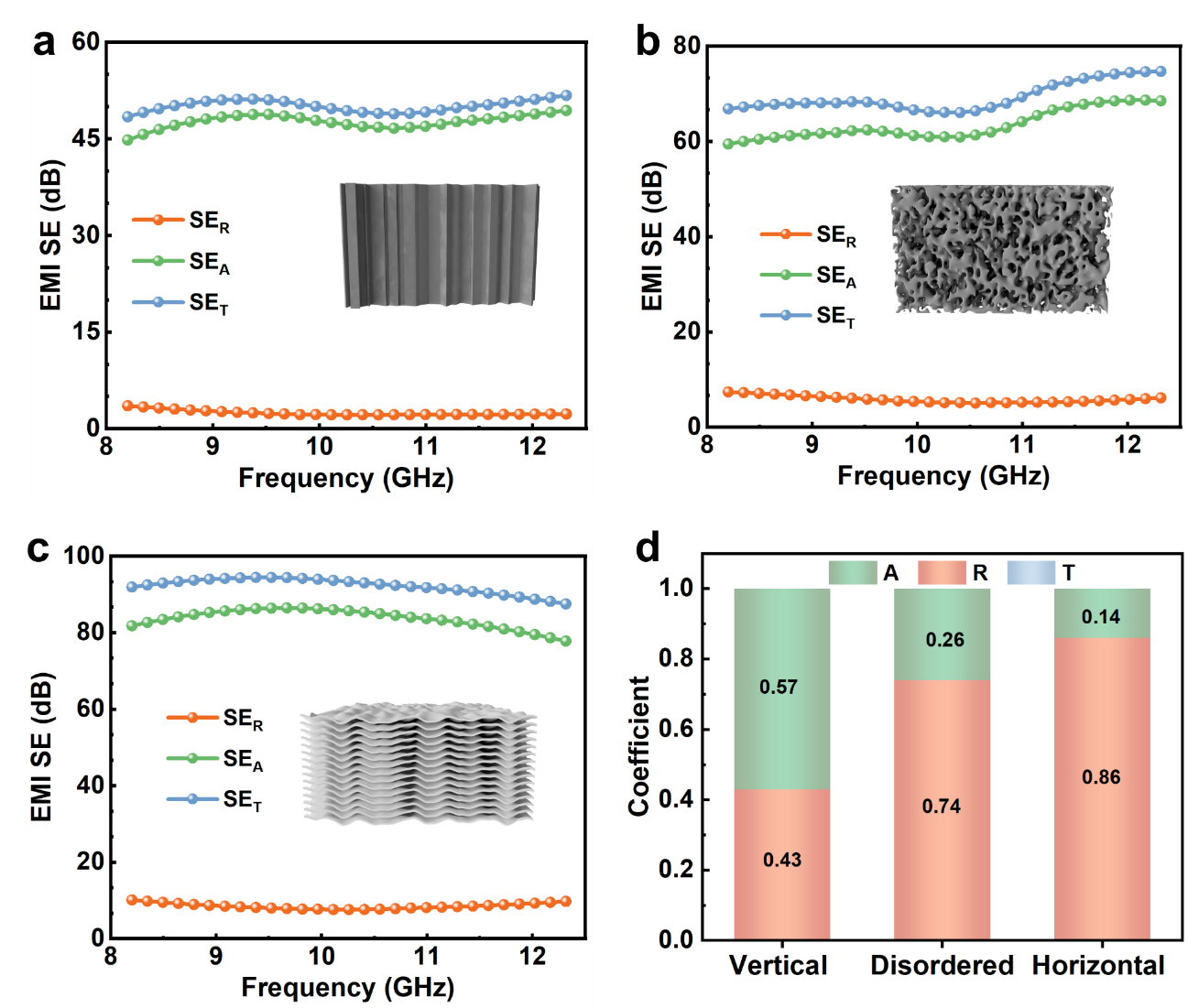


Fig. S13 EMI SE_T_, SE_R_, and SE_A_ of the (a) vertical structure C-MXene/PI aerogel, (b) disordered structure C-MXene/PI aerogel and (c) horizontal structure C-MXene/PI aerogel. (d) Coefficients of vertical, disordered and horizontal structure C-MXene/PI aerogel


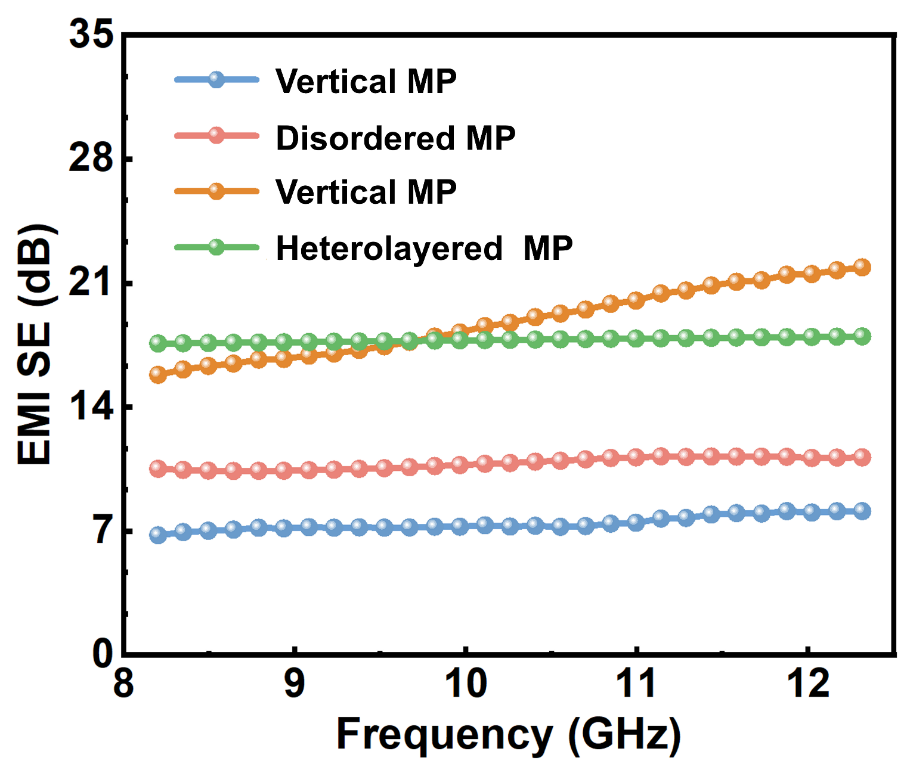


Fig. S14 EMI SE of MP with different structures


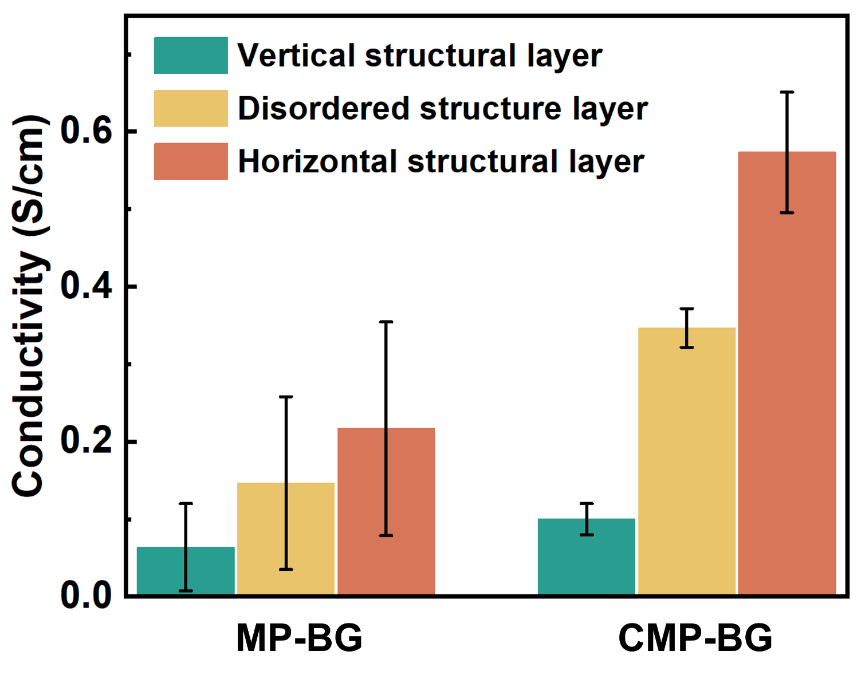


Fig. S15 The electrical conductivities of the vertical, disordered and horizontal structural layers of MP and CMP


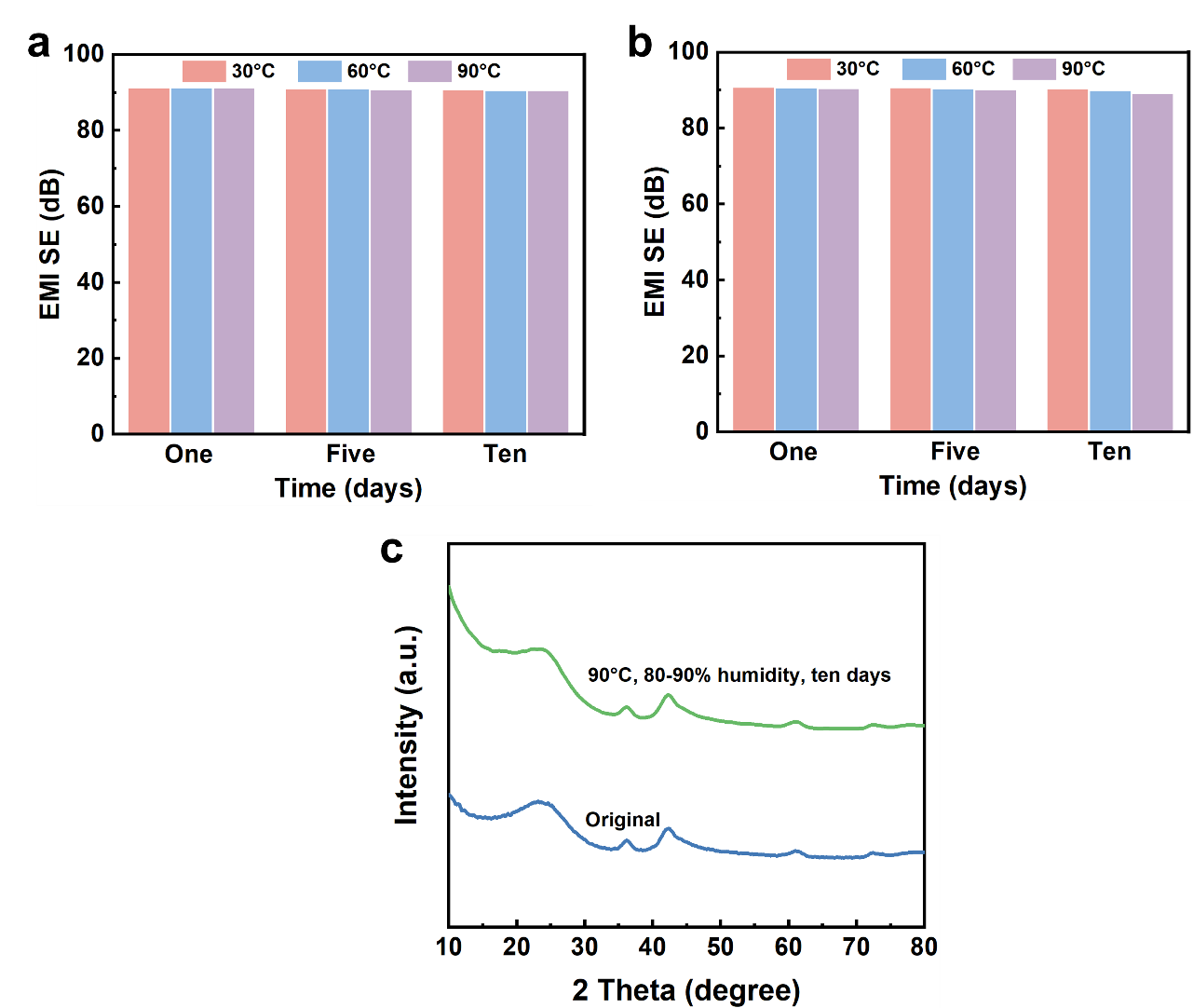


**Fig. S16** (**a**) EMI SE of CMP after exposure at 40-50% humidity and different temperatures for varying durations. (**b**) EMI SE of CMP after exposure at 80-90% humidity and different temperatures for varying durations. (**c**) XRD patterns of the pristine CMP sample and after 10 days of exposure in a damp-heat environment


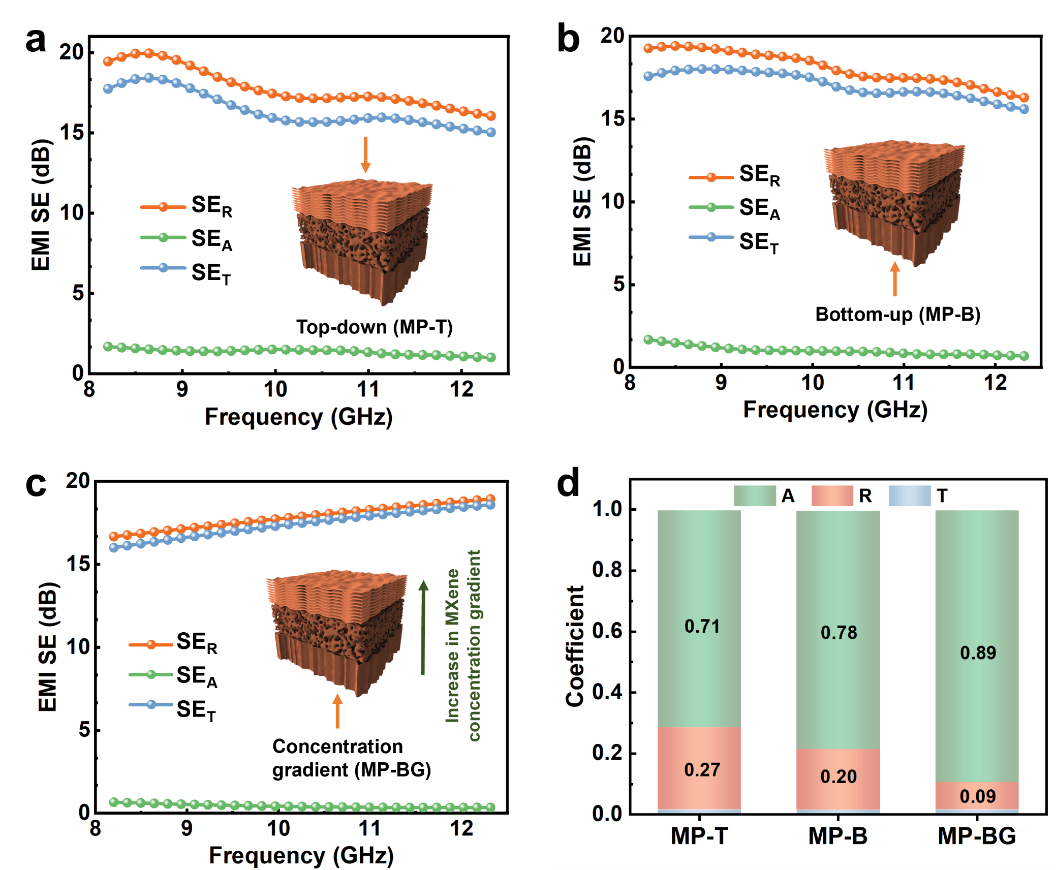


Fig. S17 EMI SE_T_, SE_R_, and SE_A_ of the (a) MP-T, (b) MP-B and (c) MP-BG. (d) Coefficients of MP-T, MP-B and MP-BG


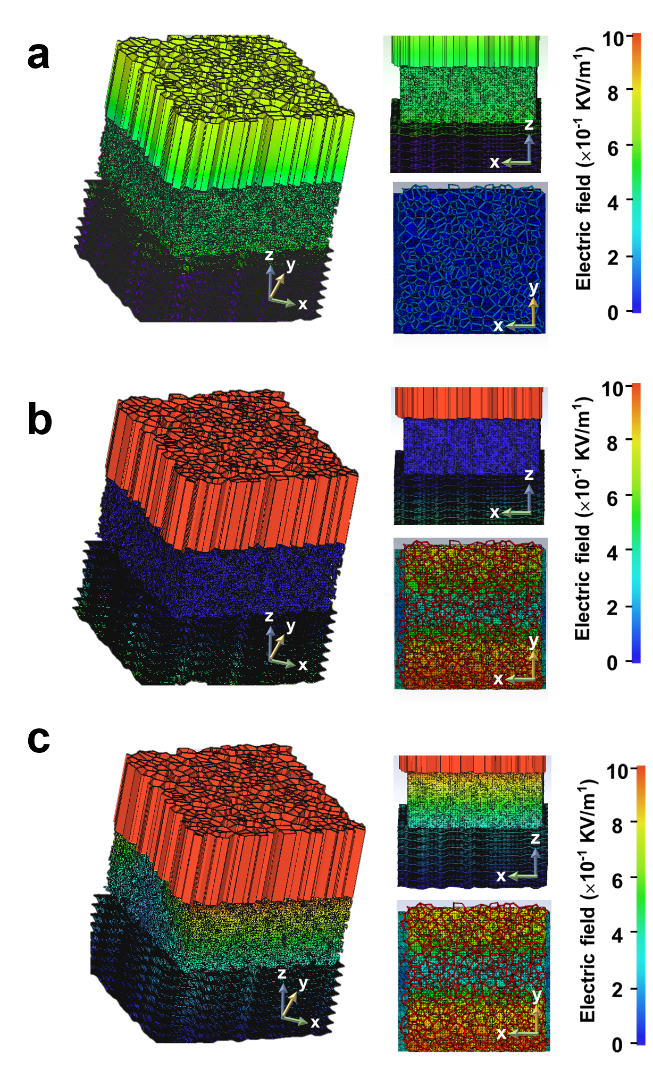


Fig. S18 The electric field (EF) distribution maps of (a) CMP-T, (b) CMP-B and (c) CMP-BG


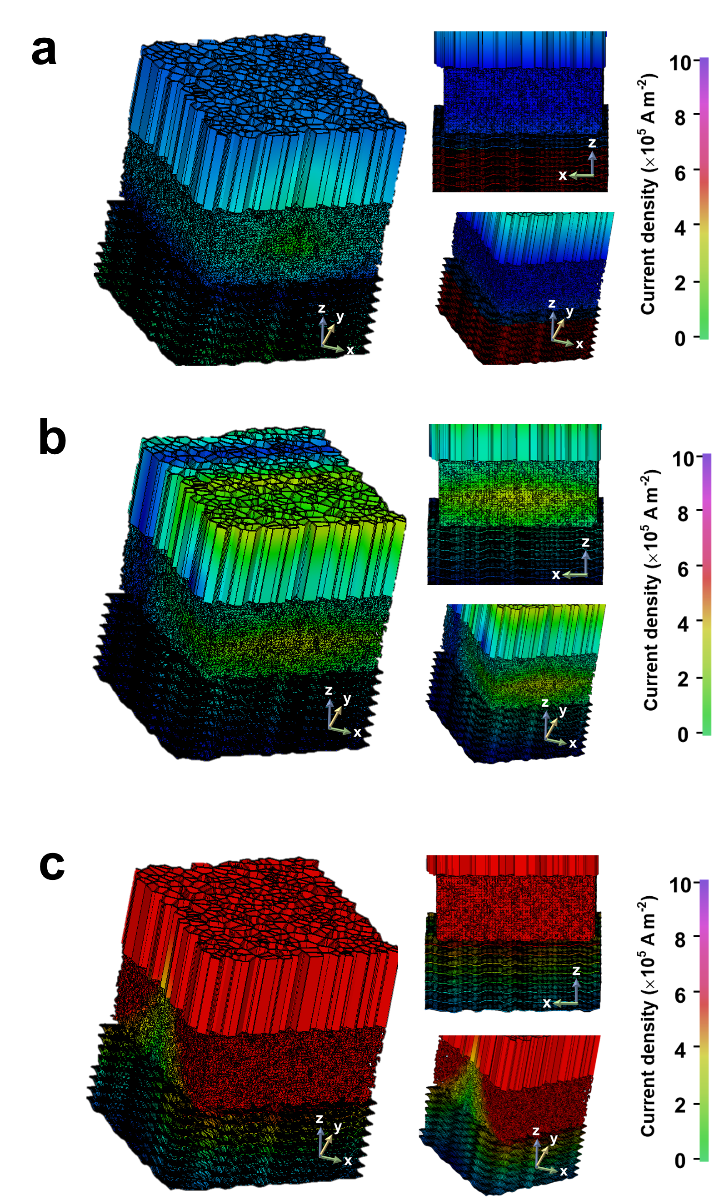


**Fig**. **S19** The electrical energy density (EED) distribution maps of (**a**) CMP-T, (**b**) CMP-B and (**c**) CMP-BG


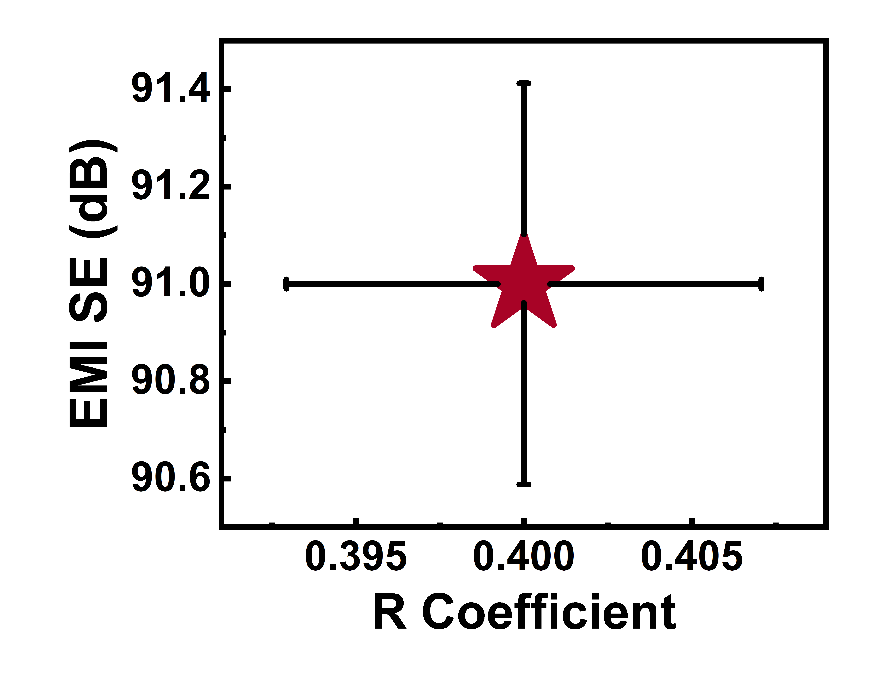


Fig. S20 The enlarged error bar illustration in Fig. 2j


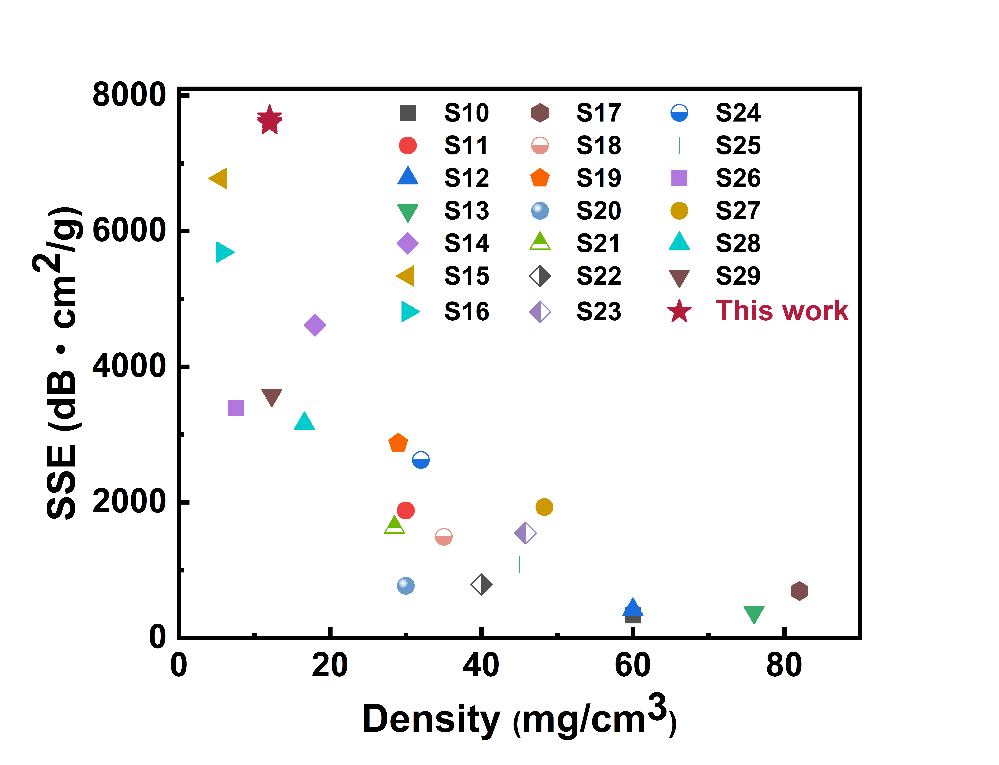


Fig. S21 Comparison of SSE and density with those of previously reported EMI shielding materials


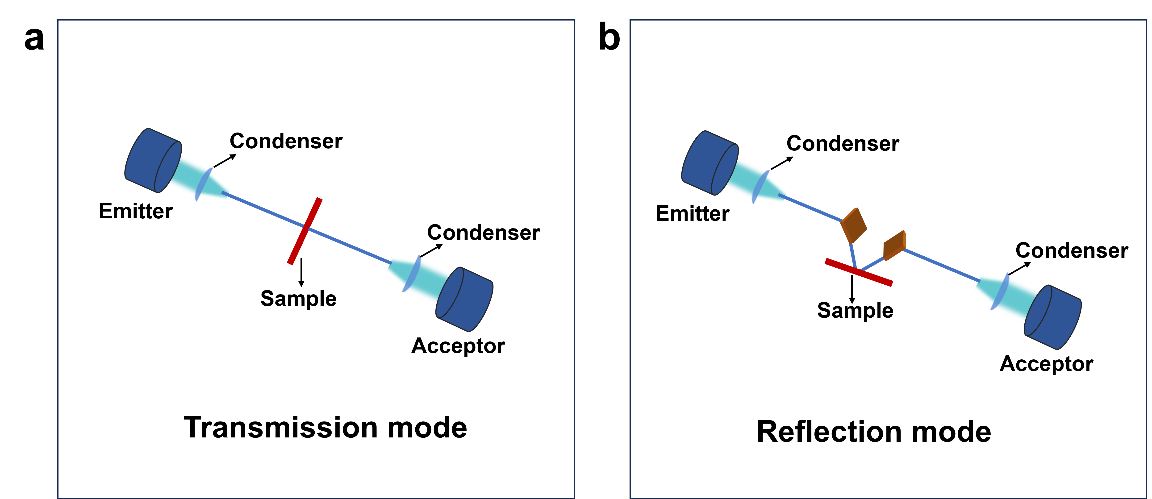


Fig. S22 The schematic diagram of (a) transmission mode and (b) reflection mode for THz time-domain spectral test


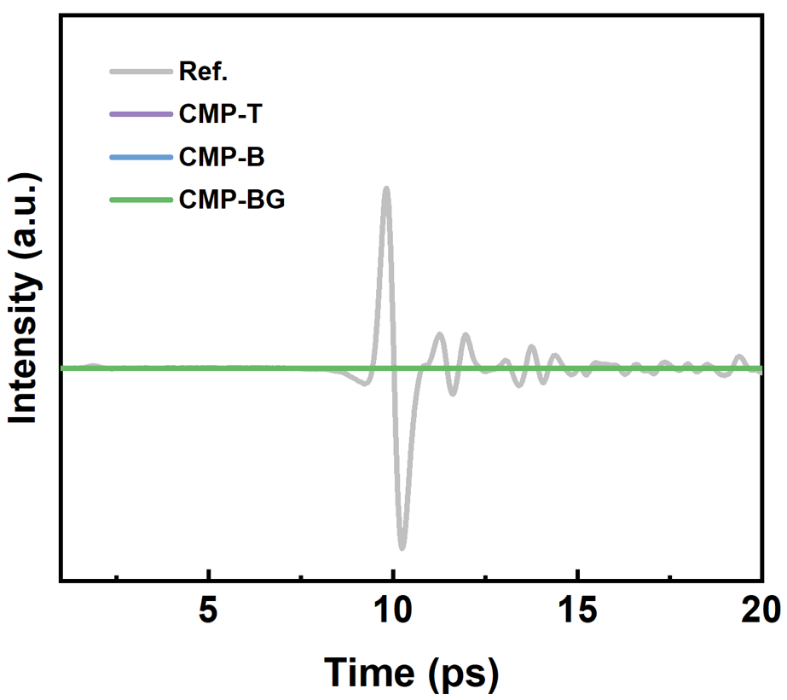


Fig. S23 The THz time-domain spectroscopy of CMP for transmission mode


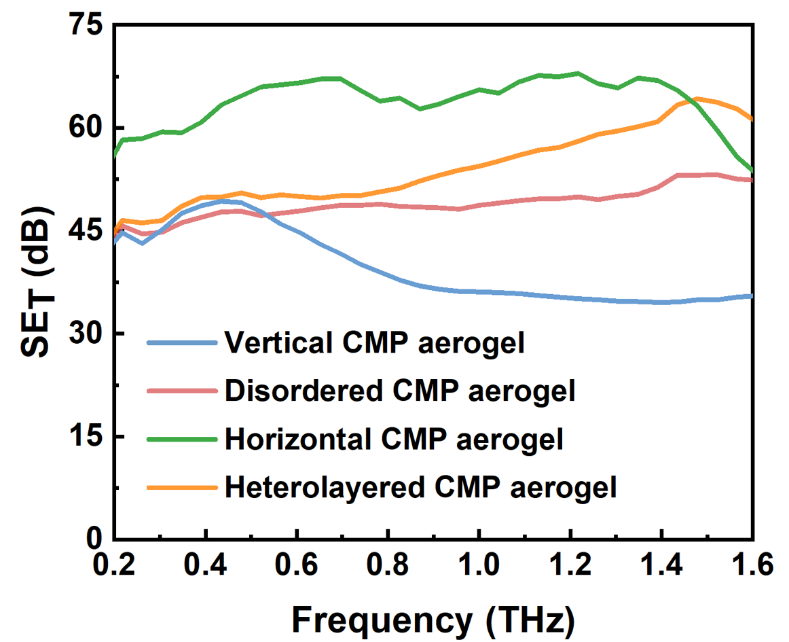


**Fig. S24** SE_T_ of CMP with different structures in a frequency range of 0.2-1.6 THz


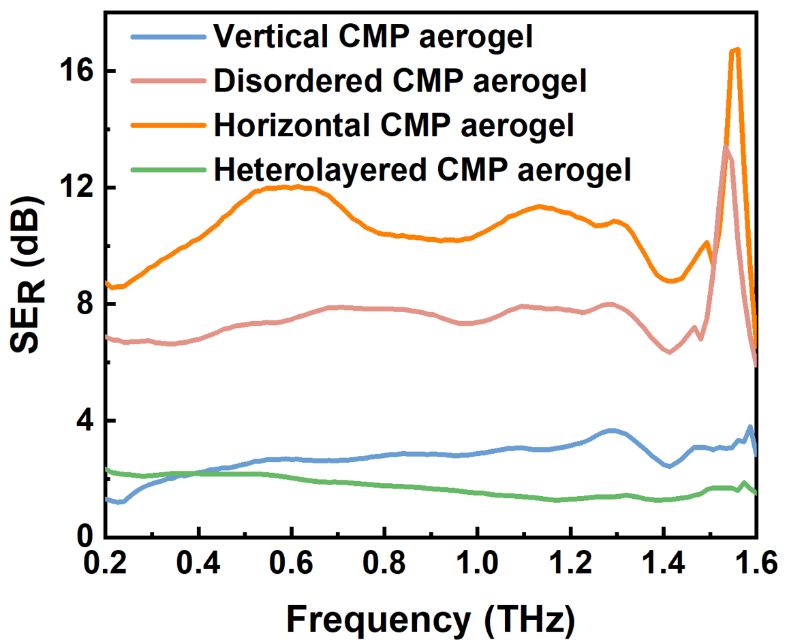


**Fig. S25** SE_R_ of different structural CMP aerogels in a frequency range of 0.2-1.6 THz


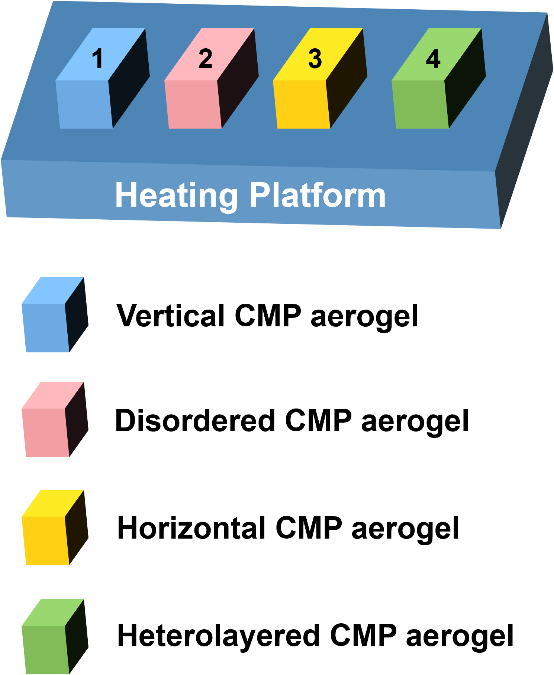


**Fig. S26** Schematic diagram of heating the sample on the heating platform


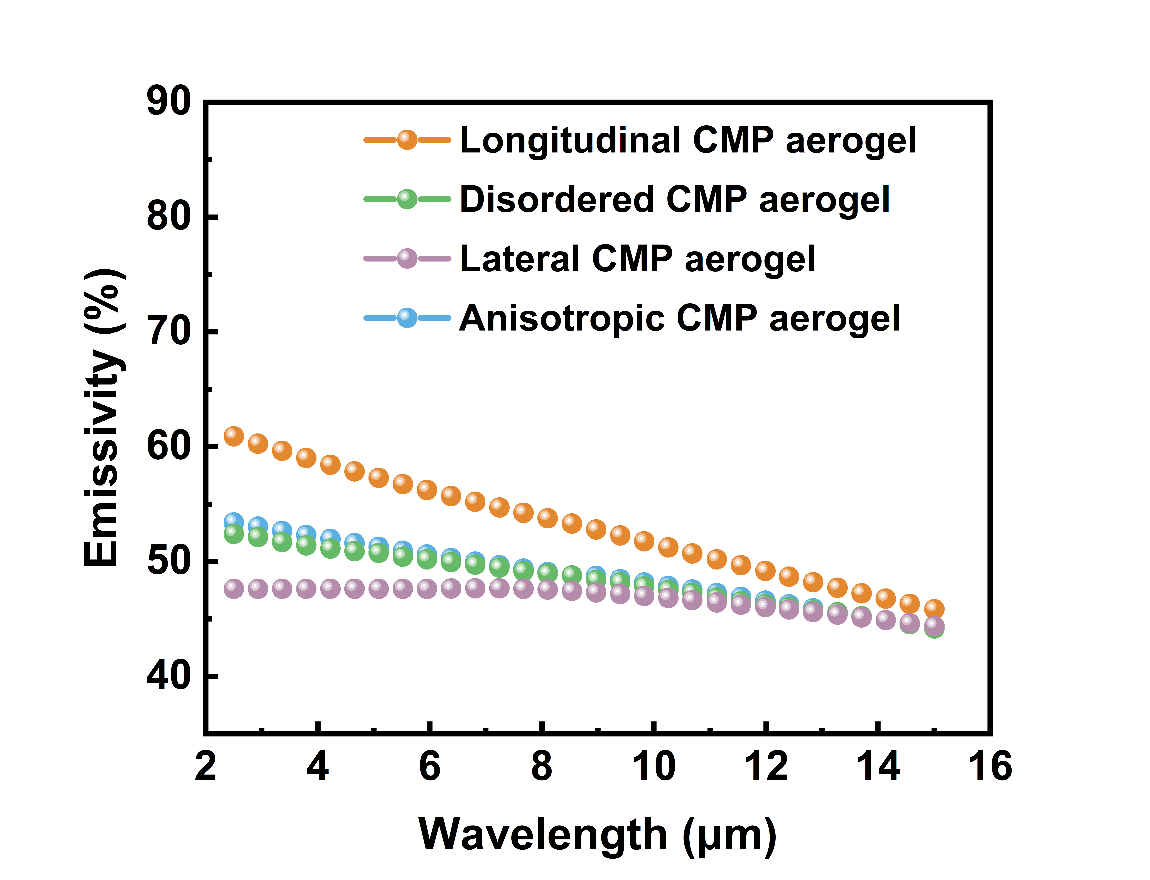


**Fig. S27** The infrared emissivity of different structured CMP in the 2-16 μm

**Table S1** The thickness of CMP aerogels with different structures

| **Sample** | **Aggregate thickness**  **(cm)** | **Vertical**  **layer thickness (cm)** | **Disordered layer thickness (cm)** | **Horizontal layer thickness (cm)** |
| --- | --- | --- | --- | --- |
| Vertical CMP aerogel | 1.00 | 1.00 | 0 | 0 |
| Disordered CMP aerogel | 1.00 | 0 | 1.00 | 0 |
| Horizontal CMP aerogel | 1.00 | 0 | 0 | 1.00 |
| Heterolayered CMP aerogel | 1.00 | 0.33 | 0.33 | 0.33 |

**Table S2** SE_T_, SE_A_ and SE_R_ average value of CMP-T, CMP-B and CMP-BG

| **Material type** | **EMI SE_T_** | **EMI SE_A_** | **EMI SE_R_** |
| --- | --- | --- | --- |
| CMP-T | 91.4 | 85.4 | 6.0 |
| CMP-B | 91.3 | 88.9 | 2.4 |
| CMP-BG | 91.0 | 88.8 | 2.2 |

**Table S3** Comparison of EMI SE and R Coefficient with those of previously reported EMI shielding materials

| **Material type** | **EMI SE (dB)** | **R** | **Refs.** |
| --- | --- | --- | --- |
| CoNi/MXene/nanocellulose | 35.1 | 0.72 | [S1] |
|  | 21.2 | 0.75 |  |
| CNF/MXene/FeCo | 58.0 | 0.51 | [S2] |
| Fe_3_O_4_/CNT/PU&MXene | 60.0 | 0.38 | [S3] |
| TCTCF | 85.0 | 0.42 | [S4] |
| AgNWs/Fe_3_O_4_@NC | 50.0 | 0.45 | [S5] |
| CNT@MXene | 23.4 | 0.37 | [S6] |
| Cellulose/biochar | 72.4 | 0.58 | [S7] |
| Carbon black/graphene/nickel polyimide | 44.0 | 0.29 | [S8] |
| HWLS/PAN/ZIF-67 | 25.2 | 0.43 | [S9] |
| **C-MXene/PI** | **91.0** | **0.40** | **This work** |

**Table S4** Comparison of SSE and density with those of previously reported EMI shielding materials

| **Material type** | **Density (mg/cm^3^)** | **EMI SE**  **(dB)** | **SSE**  **(dB•cm^2^/g)** | **Refs.** |
| --- | --- | --- | --- | --- |
| Graphene/PDMS | 60.0 | 20.0 | 333.3 | [S10] |
| Ti_3_C_2_T_x_/RGO | 30.0 | 56.4 | 1880.0 | [S11] |
| Graphene foam | 60.0 | 25.2 | 420.0 | [S12] |
| PI/ rGO | 76.0 | 28.8 | 378.9 | [S13] |
| IGA | 18.0 | 83.0 | 4611.1 | [S14] |
| Reduced  graphene aerogel | 5.5 | 30.5 | 6777.8 | [S15] |
| CNF/rGO | 5.8 | 33.0 | 5689.7 | [S16] |
| MXene (Ti_3_C_2_T_x_)/ANFs | 82.0 | 56.8 | 692.7 | [S17] |
| MXene/CMC | 35.0 | 52.2 | 1490.0 | [S18] |
| rGO/MXene | 29.0 | 83.3 | 2872.4 | [S9] |
| PLLA-MWCNT | 30.0 | 23.0 | 766.7 | [S20] |
| O-PI/CNT | 28.5 | 46.5 | 1631.6 | [S21] |
| ANF/GN | 40.0 | 31.6 | 788.8 | [S22] |
| OSG/BNNR | 45.8 | 70.9 | 1548.0 | [S23] |
| PU@PDA@Ag | 32.0 | 84.0 | 2625.0 | [S24] |
| Polyurethane/Silver Nanowire | 45.0 | 48.6 | 1080.0 | [S25] |
| Cu Nanowire  @Graphene | 7.5 | 25.4 | 3386.7 | [S26] |
| Polyimide/graphene aerogels | 25.0 | 48.3 | 1931.0 | [S27] |
| AgNW/MXene | 16.6 | 52.5 | 3162.7 | [S28] |
| PSA/AgNWs | 12.3 | 44.0 | 3580.5 | [S29] |
| **CMP-T** | **12.0** | **91.4** | **7616.7** | **This work** |
| **CMP-B** | **12.0** | **91.3** | **7680.3** | **This work** |
| **CMP-BG** | **12.0** | **91.0** | **7583.3** | **This work** |

**Table S5** The EMI SE, R and thermal conductivity of five batches of independent experiments for heterolayered CMP aerogels

| **Sample** | **EMI SE in X band (dB)** | **R in X band** | **EMI SE in THz band (dB)** | **R in THz band** | **Thermal conductivity (W/(m•K))** |
| --- | --- | --- | --- | --- | --- |
| 1 | 91.0 | 0.40 | 66.2 | 0.33 | 0.383 |
| 2 | 91.3 | 0.40 | 68.0 | 0.35 | 0.386 |
| 3 | 90.5 | 0.39 | 63.7 | 0.32 | 0.377 |
| 4 | 90.7 | 0.40 | 64.4 | 0.34 | 0.380 |
| 5 | 91.5 | 0.41 | 68.7 | 0.31 | 0.389 |

**Table S6** The R, t and $\sigma$ of CMP-T, CMP-B and CMP-BG aerogels.

| **Sample** | **Vertical layer** | | | **Disordered layer** | | | | **Horizontal layer** | | |
| --- | --- | --- | --- | --- | --- | --- | --- | --- | --- | --- |
|  | **R** | **t** | $\boldsymbol{\sigma}$ | **R** | **t** | $\boldsymbol{\sigma}$ | | **R** | **t** | $\boldsymbol{\sigma}$ |
| CMP-T | 8.9 | 0.33 | 0.34 | 8.9 | 0.33 | 0.34 | | 8.9 | 0.33 | 0.34 |
| CMP-B | 8.9 | 0.33 | 0.34 | 8.9 | 0.33 | 0.34 | | 8.9 | 0.33 | 0.34 |
| CMP-BG | 30.3 | 0.33 | 0.10 | 8.9 | 0.33 | | 0.34 | 5.3 | 0.33 | 0.57 |

**Table S7** The electromagnetic wave incidence direction and electrical conductivity of CMP-T, CMP-B and CMP-BG aerogels.

| **Sample** | **The incident direction of EW** | **Vertical**  **layer conductivity (S/cm)** | | **Disordered layer conductivity (S/cm)** | **Horizontal layer conductivity (S/cm)** |
| --- | --- | --- | --- | --- | --- |
| CMP-T | From the horizontal layer | | 0.34 | 0.34 | 0.34 |
| CMP-B | From the vertical layer | | 0.34 | 0.34 | 0.34 |
| CMP-BG | From the vertical layer | | 0.10 | 0.34 | 0.57 |

**Supplementary References**

1. T. Mai, L. Chen, Q. Liu, Z.-H. Guo, M.-G. Ma, Zeolitic imidazolate frameworks derived magnetic nanocage/MXene/nanocellulose bilayer aerogels for low reflection electromagnetic interference shielding and light-to-heat conversion. Adv. Funct. Mater. **35**(13), 2417947 (2025). <https://doi.org/10.1002/adfm.202417947>
2. M. Ma, W. Tao, X. Liao, S. Chen, Y. Shi et al., Cellulose nanofiber/MXene/FeCo composites with gradient structure for highly absorbed electromagnetic interference shielding. Chem. Eng. J. **452**, 139471 (2023). <https://doi.org/10.1016/j.cej.2022.139471>
3. J. Zhang, L. Zeng, X. Liu, D. Zhang, A. Gao et al., Lattice-filler dual-gradient and hierarchical porous architectures customized by multiple-nozzle 3D printing towards excellent absorption-dominant electromagnetic interference shielding. Compos. Sci. Technol. **262**, 111058 (2025). <https://doi.org/10.1016/j.compscitech.2025.111058>
4. L. Wang, J. Men, W. Ren, Z. Yang, Y. Gao et al., Low-reflection electromagnetic interference shielding composite foams with asymmetric structure towards infrared camouflage and response switching. J. Colloid Interface Sci. **697**, 137941 (2025). <https://doi.org/10.1016/j.jcis.2025.137941>
5. M. Sun, Z. Wang, J. Xiao, X. Tian, X. Ma et al., AgNWs/Fe_3_O_4_@NC conductive network hierarchical assembly to prepare flexible EMI shielding textile. Small **20**(14), 2304622 (2024). <https://doi.org/10.1002/smll.202304622>
6. W. Feng, L. Zou, C. Lan, S. E, X. Pu, Core-sheath CNT@MXene fibers toward absorption-dominated electromagnetic interference shielding fabrics. Adv. Fiber Mater. **6**(5), 1657–1668 (2024). <https://doi.org/10.1007/s42765-024-00452-2>
7. S. Li, Y. Du, H. Ye, J. Wu, Y. Wang et al., All-natural self-bonded biocomposite providing superior electromagnetic interference shield performance with effective absorption. Adv. Funct. Mater. **34**(41), 2406282 (2024). <https://doi.org/10.1002/adfm.202406282>
8. Y. Wang, Y. Zhong, J. Kang, B. Zhang, Z. Ma et al., Multifunctional rigid polyimide foams with outstanding EMI shielding and wave absorption *via* densification strategy. J. Mater. Sci. Technol. **227**, 155–163 (2025). <https://doi.org/10.1016/j.jmst.2024.12.021>
9. D. Gao, S. Guo, Y. Zhou, B. Lyu, X. Li et al., Absorption-dominant, low-reflection multifunctional electromagnetic shielding material derived from hydrolysate of waste leather scraps. ACS Appl. Mater. Interfaces **14**(33), 38077–38089 (2022). <https://doi.org/10.1021/acsami.2c10787>
10. Z. Chen, C. Xu, C. Ma, W. Ren, H.-M. Cheng, Lightweight and flexible graphene foam composites for high-performance electromagnetic interference shielding. Adv. Mater. **25**(9), 1296–1300 (2013). <https://doi.org/10.1002/adma.201204196>
11. S. Zhao, H.-B. Zhang, J.-Q. Luo, Q.-W. Wang, B. Xu et al., Highly electrically conductive three-dimensional Ti_3_C_2_T*_x_* MXene/reduced graphene oxide hybrid aerogels with excellent electromagnetic interference shielding performances. ACS Nano **12**(11), 11193–11202 (2018). <http://doi.org/10.1021/acsnano.8b05739>
12. B. Shen, Y. Li, D. Yi, W. Zhai, X. Wei et al., Microcellular graphene foam for improved broadband electromagnetic interference shielding. Carbon **102**, 154–160 (2016). <https://doi.org/10.1016/j.carbon.2016.02.040>
13. 13 Z. Yu, T. Dai, S. Yuan, H. Zou, P. Liu, Electromagnetic interference shielding performance of anisotropic polyimide/graphene composite aerogels. ACS Appl. Mater. Interfaces **12**(27), 30990–31001 (2020). <https://doi.org/10.1021/acsami.0c07122>
14. J. Liu, Y. Liu, H.-B. Zhang, Y. Dai, Z. Liu et al., Superelastic and multifunctional graphene-based aerogels by interfacial reinforcement with graphitized carbon at high temperatures. Carbon **132**, 95–103 (2018). <https://doi.org/10.1016/j.carbon.2018.02.026>
15. S. Bi, L. Zhang, C. Mu, M. Liu, X. Hu, Electromagnetic interference shielding properties and mechanisms of chemically reduced graphene aerogels. Appl. Surf. Sci. **412**, 529–536 (2017). <https://doi.org/10.1016/j.apsusc.2017.03.293>
16. M. Li, F. Han, S. Jiang, M. Zhang, Q. Xu et al., Lightweight cellulose nanofibril/reduced graphene oxide aerogels with unidirectional pores for efficient electromagnetic interference shielding. Adv. Mater. Interfaces **8**(24), 2101437 (2021). <https://doi.org/10.1002/admi.202101437>
17. Z. Lu, F. Jia, L. Zhuo, D. Ning, K. Gao et al., Micro-porous MXene/Aramid nanofibers hybrid aerogel with reversible compression and efficient EMI shielding performance. Compos. Part B Eng. **217**, 108853 (2021). <https://doi.org/10.1016/j.compositesb.2021.108853>
18. Y. Cheng, W. Zhu, X. Lu, C. Wang, Lightweight and flexible MXene/carboxymethyl cellulose aerogel for electromagnetic shielding, energy harvest and self-powered sensing. Nano Energy **98**, 107229 (2022). <https://doi.org/10.1016/j.nanoen.2022.107229>
19. X. Zheng, J. Tang, P. Wang, Z. Wang, L. Zou et al., Interfused core-shell heterogeneous graphene/MXene fiber aerogel for high-performance and durable electromagnetic interference shielding. J. Colloid Interface Sci. **628**, 994–1003 (2022). <https://doi.org/10.1016/j.jcis.2022.08.019>
20. T. Kuang, L. Chang, F. Chen, Y. Sheng, D. Fu et al., Facile preparation of lightweight high-strength biodegradable polymer/multi-walled carbon nanotubes nanocomposite foams for electromagnetic interference shielding. Carbon **105**, 305–313 (2016). <https://doi.org/10.1016/j.carbon.2016.04.052>
21. S. Liu, Q. Xu, Y. Bai, X. Wang, X. Liu et al., Toward strong X-band-electromagnetic-wave-absorbing materials: polyimide/carbon nanotube composite aerogel with radial needle-like porous structure. J. Mater. Chem. A **10**(47), 25140–25147 (2022). <http://doi.org/10.1039/d2ta05845f>
22. N. Luo, Y.-Y. Zhang, H. Zhang, T.-L. Liu, Y. Wang et al., Electromagnetic interference shielding performance of lightweight aramid nanofiber/graphene composite aerogels. J. Mater. Chem. A **12**(17), 10359–10368 (2024). <http://doi.org/10.1039/d3ta07473k>
23. L. Feng, P. Wei, Q. Song, J. Zhang, Q. Fu et al., Superelastic, highly conductive, superhydrophobic, and powerful electromagnetic shielding hybrid aerogels built from orthogonal graphene and boron nitride nanoribbons. ACS Nano **16**(10), 17049–17061 (2022). <https://doi.org/10.1021/acsnano.2c07187>
24. C. Liang, Y. Liu, Y. Ruan, H. Qiu, P. Song et al., Multifunctional sponges with flexible motion sensing and outstanding thermal insulation for superior electromagnetic interference shielding. Compos. Part A Appl. Sci. Manuf. **139**, 106143 (2020). <https://doi.org/10.1016/j.compositesa.2020.106143>
25. Z. Zeng, M. Chen, Y. Pei, S.I. Seyed Shahabadi, B. Che et al., Ultralight and flexible polyurethane/silver nanowire nanocomposites with unidirectional pores for highly effective electromagnetic shielding. ACS Appl. Mater. Interfaces **9**(37), 32211–32219 (2017). <http://doi.org/10.1021/acsami.7b07643>
26. S. Wu, M. Zou, Z. Li, D. Chen, H. Zhang et al., Robust and stable Cu Nanowire@Graphene core–shell aerogels for ultraeffective electromagnetic interference shielding. Small **14**(23), 1800634 (2018). <https://doi.org/10.1002/smll.201800634>
27. Y. Zhao, H. Chen, S. Qiao, Z. Wang, J. Yan, Hierarchically porous polyimide/graphene aerogels with superior compressibility and electromagnetic interference shielding performance. J. Mater. Chem. A **13**(28), 22613–22620 (2025). <http://doi.org/10.1039/d5ta02068a>
28. C. Weng, G. Wang, Z. Dai, Y. Pei, L. Liu et al., Buckled AgNW/MXene hybrid hierarchical sponges for high-performance electromagnetic interference shielding. Nanoscale **11**(47), 22804–22812 (2019). <https://doi.org/10.1039/c9nr07988b>
29. J. Wang, Q. Zhang, S. Zhang, R. Liu, Y. Zhang et al., Multifunctional Polysulfonamide/AgNWs aerogel for electromagnetic interference shielding. Compos. Commun. **56**, 102346 (2025). <https://doi.org/10.1016/j.coco.2025.102346>
